# Serial H&E Pathology Reveals Progression‐Associated Nuclear Size Drift as a Longitudinal Digital Readout of Bladder Cancer Evolution

**DOI:** 10.1002/advs.78299

**Published:** 2026-10-14

**Authors:** Xing Liu, Zhen‐Duo Shi, Gong‐Cheng Wang, Jian‐Ming Wang, Ying Liu, Yi Gong, Xuan Lin, Di Wu, Shi‐Chao Zhuo, Han Han, Yi‐Bing Shi, Fan‐Lai Meng, Yu Zhang, Zhi‐Gang Jia, Cong‐Hui Han

**Affiliations:** ^1^ Department of Urology Xuzhou Central Hospital School of Medicine Southeast University Xuzhou Jiangsu China; ^2^ Xuzhou Central Hospital Xuzhou Jiangsu China; ^3^ School of Life Sciences Jiangsu Normal University Xuzhou China; ^4^ Department of Urology The Affiliated Huai'an No.1 People's Hospital of Nanjing Medical University Huaian Jiangsu China; ^5^ Linyi People's Hospital Shandong Second Medical University Linyi Shandong China; ^6^ Jiangsu Provincial Engineering Research Center of Cancer Cell Therapy and Translational Medicine Xuzhou Central Hospital Southeast University Xuzhou Jiangsu China; ^7^ School of Clinical Medicine Shandong Second Medical University Weifang Shandong China; ^8^ The Huaian Clinical College of Xuzhou Medical University Huaian Jiangsu China; ^9^ Department of Pathology Xuzhou Central Hospital Southeast University Xuzhou Jiangsu China; ^10^ Department of Information Xuzhou Central Hospital Southeast University Xuzhou Jiangsu China; ^11^ Department of Radiology Xuzhou Central Hospital Southeast University Xuzhou Jiangsu China; ^12^ Department of Pathology The Affiliated Suqian Hospital of Xuzhou Medical University Suqian Jiangsu China; ^13^ State Key Laboratory of Digital Medical Engineering Jiangsu Key Laboratory for Biomaterials and Devices School of Biological Science and Medical Engineering Southeast University Nanjing China; ^14^ School of Mathematics and Statistics and Jiangsu Key Laboratory of Education Big Data Science and Engineering Jiangsu Normal University Xuzhou Jiangsu China

**Keywords:** bladder cancer, computational pathology, hematoxylin and eosin, longitudinal digital pathology, nuclear size drift, tumor evolution, whole‐slide imaging

## Abstract

Serial pathological specimens are routinely generated during bladder cancer surveillance, but their temporal information remains underused in computational pathology. This study develops a longitudinal digital pathology framework to quantify direction‐preserving nuclear morphological change within the same patient. Paired hematoxylin and eosin whole‐slide images are organized as T0, the earlier specimen, and T1, the later specimen. Signed Δ features, defined as T1−T0, are extracted across nuclear size, shape regularity, local cellularity, and spatial organization. After quality control, the discovery cohort includes 86 pairs from 86 patients, and the independent confirmation cohort includes 96 pairs from 96 patients. A separate four‐center resource of 1977 whole‐slide images from 1660 patients provides a static contextual baseline. Several nuclear features showed the same direction of association with progression across the two cohorts. Nuclear size‐related change was most prominent in the independent confirmation cohort, particularly for equivalent nuclear diameter and nuclear area. Spatial analysis shows a broader upward distributional shift in nuclear area in progression pairs. Static foundation‐model representations show variable locked cross‐center transferability, whereas Ki‐67 immunohistochemistry and TCGA‐BLCA transcriptomic analyses provide complementary tissue‐level and molecular context. Routine serial hematoxylin and eosin specimens may therefore provide interpretable, direction‐preserving digital readouts of bladder cancer evolution.

## Graphical abstract

Routine serial H&E specimens from bladder cancer are converted into paired T0/T1 digital pathology readouts of tumor evolution. Progression is associated with longitudinal nuclear drift, with nuclear size‐related changes most evident in the independent confirmation cohort, alongside a broader spatial burden of increased nuclear area and biological support from Ki‐67 immunohistochemistry and TCGA transcriptomic anchoring.

**Abbreviations**
AUC: Area under the receiver operating characteristic curve
BCG: Bacillus Calmette–Guérin
CI: Confidence interval
CIS: Carcinoma in situ
CV: Coefficient of variation
FDR: False discovery rate
H&E: Hematoxylin and eosin
HER2: Human epidermal growth factor receptor 2
HR: Hazard ratio
IFN‐γ: Interferon‐gamma
IHC: Immunohistochemistry
IQR: Interquartile range
MIBC: Muscle‐invasive bladder cancer
NMIBC: Non‐muscle‐invasive bladder cancer
QC: Quality control
RFS: Recurrence‐free survival
ROC: Receiver operating characteristic
SMD: Standardized mean difference
TCGA: The Cancer Genome Atlas
TURBT: Transurethral resection of bladder tumor
WSI: Whole‐slide image

## 1 Introduction

Bladder cancer is one of the few solid tumors in which repeated pathological sampling is built into routine care [1, 2, 3]. Patients, especially those initially diagnosed with non‐muscle‐invasive bladder cancer, often undergo serial resections or biopsies during surveillance, recurrence evaluation, and subsequent treatment [4]. These specimens are usually read as separate diagnostic events: a tumor is assigned a grade, a stage, and other pathological descriptors at each time point. What is rarely measured is the direction of change between specimens from the same patient. A recurrent tumor that looks aggressive at one time point is not necessarily the same biological situation as a tumor that has gradually shifted toward a more aggressive morphology over time [5]. This distinction is clinically familiar but remains poorly quantified in routine pathology.

Digital pathology offers a way to revisit this problem. More broadly, artificial intelligence and deep learning have expanded the ability to extract biomarker‐relevant information from routine histology images [6, 7]. However, most computational pathology studies in bladder cancer still treat whole‐slide images as static samples [8, 9]. In bladder cancer, existing models have been used to classify tumor type, estimate grade or stage, infer molecular phenotype, predict recurrence, or stratify survival risk from one specimen or one slide‐level representation [8, 10, 11]. Large‐scale weakly supervised WSI studies in computational pathology have also shown that clinically relevant slide‐level models can be trained without exhaustive pixel‐level annotation [12]. These studies indicate that H&E images carry measurable information about nuclear morphology, tissue architecture, and the local tumor microenvironment. At the same time, a single slide captures only a sampled state of the tumor. It is affected by tissue region, tumor content, staining, scanner characteristics, and institutional practice [13, 14]. More importantly, it does not say whether the tumor is moving toward or away from a more aggressive pathological state.

A longitudinal, within‐patient design addresses a different question [2, 15]. Instead of asking whether one WSI contains risk‐related information, it asks whether the morphology of the same patient's tumor changes in a measurable and directionally meaningful way. In such a design, the earlier pathological specimen can be treated as T0, and the later specimen as T1, and signed T1−T0 differences can be analyzed as longitudinal morphological drift. This approach is particularly suited to bladder cancer because serial specimens are routinely generated as part of clinical management [1, 2]. It also avoids treating repeated samples from the same patient as unrelated observations, a limitation that is difficult to overcome in conventional cross‐sectional WSI modeling.

Among possible histological readouts, nuclear phenotype is a natural starting point [10, 16, 17]. Nuclear enlargement, altered nuclear contour, and changes in cellular organization are central to pathological assessment of urothelial carcinoma, and they can be quantified from H&E whole‐slide images at scale [10, 16, 17, 18]. Still, most available evidence linking nuclear morphology to aggressive tumor behavior comes from cross‐sectional comparisons [10, 18, 19]. It remains unclear whether progression in bladder cancer is accompanied by a reproducible within‐patient nuclear shift, whether such a shift can be observed across independent institutional cohorts, and which nuclear domain carries the most stable signal. This is important because a broad “morphological drift” claim is not enough; a useful digital pathology readout should be traceable to interpretable tissue features and should survive basic tests of reproducibility.

Here, we develop a longitudinal digital pathology framework for converting routine serial H&E whole‐slide images into signed, direction‐preserving readouts of intrapatient tumor evolution. Using two independent paired bladder cancer cohorts, we defined the earlier specimen as T0 and the later specimen as T1, then quantified pair‐level T1−T0 nuclear features across nuclear size, shape regularity, local cellularity, and spatial organization. Center 1 was used as the discovery cohort and Center 2 as the independent confirmation cohort. To place the paired analysis in a broader digital pathology context, we also analyzed a four‐center institutional WSI resource using pretrained pathology foundation models as static contextual baselines [20, 21, 22, 23]. Ki‐67 immunohistochemistry and TCGA‐BLCA transcriptomic data were used as tissue‐level and molecular anchoring layers rather than as longitudinal validation [24, 25, 26, 27, 28]. The final biomarker interpretation therefore focused on nuclear size‐related drift, which was most clearly observed in the independent confirmation cohort and remained directly interpretable in relation to conventional histopathological assessment.

## 2 Results

### 2.1 Construction of Paired Longitudinal Pathology Cohorts

From patients with eligible serial pathological specimens in Centers 1 and 2, we constructed two independent paired pathology cohorts to examine intrapatient morphological change in bladder cancer. For each patient, the earlier pathological specimen was assigned as T0 and the later specimen as T1, with the corresponding H&E whole‐slide images used for feature extraction. Progression status was assigned at the pair level. For each feature, Δ was defined as the T1 value minus the corresponding T0 value, so positive and negative values preserved the direction of intrapatient morphological change.

After pair construction and quality control, Center 1 retained 86 unique patients contributing 86 valid T0–T1 pairs, including 35 progression and 51 non‐progression pairs. Center 2 retained 96 unique patients contributing 96 valid T0–T1 pairs, including 38 progression and 58 non‐progression pairs (Figure 1B,C; Table 1 and Tables S1–S3). No patient contributed more than one pair to the final longitudinal analysis. Pair‐level QC annotations were kept for sensitivity analyses rather than used as automatic exclusion criteria.

**FIGURE 1:**
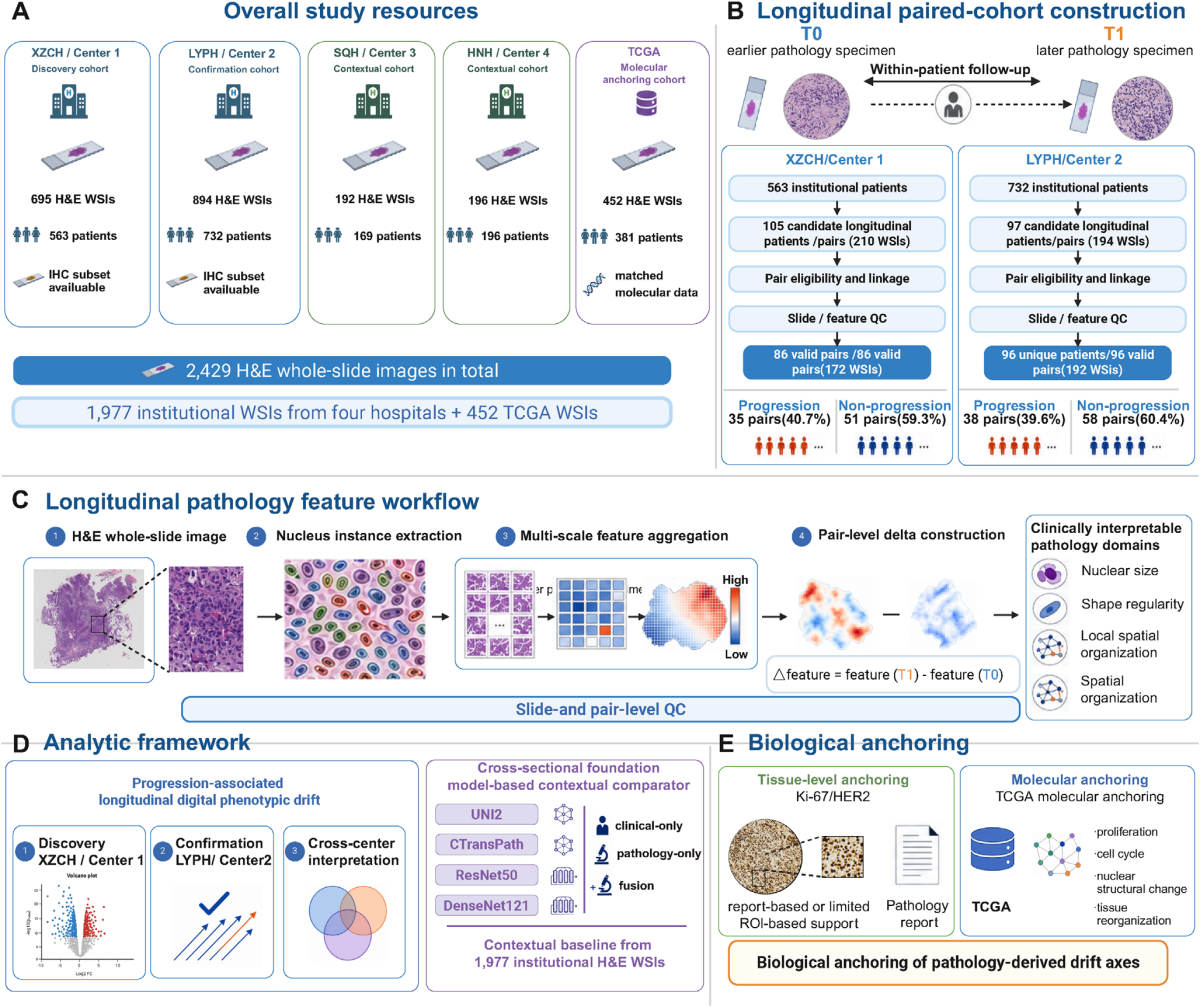
Study architecture and longitudinal digital pathology framework. (A) Overall study resources, including the four‐center institutional H&E WSI resource used for cross‐sectional contextual modeling and TCGA‐BLCA used for molecular anchoring. (B) Construction of the longitudinal paired cohorts from patients with eligible serial pathological specimens in Centers 1 and 2. After eligibility assessment, pair linkage, and quality control, 86 unique patients contributing 86 valid T0–T1 pairs were retained in Center 1 and 96 unique patients contributing 96 valid pairs were retained in Center 2. Each patient contributed one pair. (C) Longitudinal pathology feature workflow from H&E WSI processing and nuclear instance extraction through multi‐scale aggregation to signed T1−T0 feature construction across interpretable pathology domains. (D) Parallel analytical framework comprising the longitudinal discovery‐confirmation analysis and the cross‐sectional foundation‐model contextual benchmark. (E) Tissue‐level and molecular anchoring using immunohistochemistry and TCGA‐BLCA, respectively.

**TABLE 1:** Baseline clinicopathological characteristics of the institutional cohorts.

Characteristic	Category	Overall institutional cohort	XZCH/Center 1 discovery	LYPH/Center 2 confirmation	SQH/Center 3 contextual	HNH/Center 4 contextual
H&E whole‐slide images	Slide‐level	1977	695	894	192	196
Patients	Patient‐level	1660	563	732	169	196
Age, years	Median (IQR)	69.0 (61.0–76.0)	69.0 (62.0–78.0)	68.0 (60.8–75.0)	70.0 (62.0–76.0)	69.0 (61.0–76.2)
Age	<65 years	579 (34.9)	188 (33.4)	263 (35.9)	64 (37.9)	64 (32.7)
	≥65 years	1081 (65.1)	375 (66.6)	469 (64.1)	105 (62.1)	132 (67.3)
Sex	Male	1430 (86.1)	477 (84.7)	628 (85.8)	162 (95.9)	163 (83.2)
	Female	230 (13.9)	86 (15.3)	104 (14.2)	7 (4.1)	33 (16.8)
Presentation	Primary	1423 (85.7)	430 (76.4)	726 (99.2)	124 (73.4)	143 (73.0)
	Recurrent	237 (14.3)	133 (23.6)	6 (0.8)	45 (26.6)	53 (27.0)
Tumor number	Single	991 (59.7)	317 (56.3)	456 (62.3)	101 (59.8)	117 (59.7)
	Multiple	669 (40.3)	246 (43.7)	276 (37.7)	68 (40.2)	79 (40.3)
Tumor size	≤3 cm	1205 (72.6)	400 (71.0)	536 (73.2)	110 (65.1)	159 (81.1)
	>3 cm	455 (27.4)	163 (29.0)	196 (26.8)	59 (34.9)	37 (18.9)
Grade	Low grade	558 (33.6)	175 (31.1)	234 (32.0)	18 (10.7)	131 (66.8)
	High grade	1102 (66.4)	388 (68.9)	498 (68.0)	151 (89.3)	65 (33.2)
T stage	Ta/Tis	886 (53.4)	337 (59.9)	342 (46.7)	73 (43.2)	134 (68.4)
	T1	538 (32.4)	161 (28.6)	291 (39.8)	25 (14.8)	61 (31.1)
	≥T2	236 (14.2)	65 (11.5)	99 (13.5)	71 (42.0)	1 (0.5)
Disease category	NMIBC	1413 (85.1)	493 (87.6)	631 (86.2)	94 (55.6)	195 (99.5)
	MIBC	247 (14.9)	70 (12.4)	101 (13.8)	75 (44.4)	1 (0.5)
Concomitant CIS	Absent	1615 (97.3)	561 (99.6)	691 (94.4)	167 (98.8)	196 (100.0)
	Present	45 (2.7)	2 (0.4)	41 (5.6)	2 (1.2)	0 (0.0)
Lymphovascular invasion	Absent	1593 (96.0)	541 (96.1)	708 (96.7)	148 (87.6)	196 (100.0)
	Present	67 (4.0)	22 (3.9)	24 (3.3)	21 (12.4)	0 (0.0)
Perineural invasion	Absent	1618 (97.5)	553 (98.2)	725 (99.0)	144 (85.2)	196 (100.0)
	Present	42 (2.5)	10 (1.8)	7 (1.0)	25 (14.8)	0 (0.0)
Tumor location	Right wall	332 (20.0)	56 (9.9)	232 (31.7)	16 (9.5)	28 (14.3)
	Left wall	330 (19.9)	62 (11.0)	214 (29.2)	24 (14.2)	30 (15.3)
	Anterior wall	95 (5.7)	39 (6.9)	18 (2.5)	13 (7.7)	25 (12.8)
	Posterior wall	136 (8.2)	72 (12.8)	18 (2.5)	12 (7.1)	34 (17.3)
	Dome	82 (4.9)	46 (8.2)	15 (2.0)	6 (3.6)	15 (7.7)
	Trigone	131 (7.9)	90 (16.0)	16 (2.2)	12 (7.1)	13 (6.6)
	Bladder neck	52 (3.1)	16 (2.8)	22 (3.0)	7 (4.1)	7 (3.6)
	Multifocal/multiple sites	502 (30.2)	182 (32.3)	197 (26.9)	79 (46.7)	44 (22.4)
Surgery type	TURBT	1381 (83.2)	461 (81.9)	639 (87.3)	103 (60.9)	178 (90.8)
	Radical cystectomy	208 (12.5)	75 (13.3)	64 (8.7)	55 (32.5)	14 (7.1)
	Partial cystectomy	69 (4.2)	25 (4.4)	29 (4.0)	11 (6.5)	4 (2.0)
	Other/unknown	2 (0.1)	2 (0.4)	0 (0.0)	0 (0.0)	0 (0.0)
Postoperative treatment	None	190 (11.4)	74 (13.1)	93 (12.7)	23 (13.6)	0 (0.0)
	Intravesical chemotherapy	1260 (75.9)	405 (71.9)	576 (78.7)	100 (59.2)	179 (91.3)
	BCG	11 (0.7)	3 (0.5)	5 (0.7)	0 (0.0)	3 (1.5)
	Systemic chemotherapy/immunotherapy	199 (12.0)	81 (14.4)	58 (7.9)	46 (27.2)	14 (7.1)
Recurrence	No	1351 (81.4)	479 (85.1)	587 (80.2)	133 (78.7)	152 (77.6)
	Yes	309 (18.6)	84 (14.9)	145 (19.8)	36 (21.3)	44 (22.4)
RFS time, months	Median (IQR)	32.0 (16.0–53.0)	38.0 (21.0–56.0)	28.0 (10.0–49.0)	64.0 (19.0–82.0)	32.0 (28.0–40.2)
OS time, months	Median (IQR)	42.0 (27.0–61.0)	43.0 (28.0–59.0)	40.0 (20.0–60.0)	72.0 (57.0–88.0)	34.0 (31.0–43.0)

Note: Values are shown as median (IQR) or n (%). Patient characteristics were summarized at the patient level, and H&E WSI counts at the slide level. The longitudinal paired cohorts were derived from eligible serial‐pathology subsets of Centers 1 and 2 and are reported separately in Figure 1 and Tables S1–S3. IQR, interquartile range; H&E, hematoxylin and eosin; WSI, whole‐slide image.

In parallel, the broader institutional resource comprised 1977 institutional H&E whole‐slide images from 1660 patients across Centers 1–4 and was used as a separate static digital pathology resource. These slides provided a multicenter contextual baseline for single‐time‐point WSI modeling, but they were not used to define longitudinal drift. Thus, the four‐center WSI resource and the longitudinal paired cohorts represented complementary analytical layers rather than a single sequentially filtered cohort. The paired analyses focused on signed Δ features across nuclear size, shape regularity, local cellularity, and spatial organization (Figure 1D and Table S4).

### 2.2 Progression is Associated with Longitudinal Nuclear Drift

We also calculated an exploratory drift score from selected signed Δ features. Because feature screening and direction alignment used progression status, the score was outcome‐informed. We therefore retained it only as a descriptive supplementary analysis and did not use within‐cohort score separation as confirmatory evidence. The main longitudinal analysis focused on individual Δ features and cross‐center consistency; the exploratory score is shown only in Figure S1.

Across the 18 shared Δ features, effect sizes differed between centers (Figure 2A,B). Eleven of 18 features showed the same direction of association with progression (Figure 2C), although concordance differed by feature domain (Figure 2D). In Center 2 (38 progression and 58 non‐progression pairs), the largest effects involved nuclear size. Δ equivalent nuclear diameter mean showed Hedges' g = 0.72 (95% CI 0.29–1.14, P = 0.0004, FDR = 0.0039), and Δ nuclear area mean showed g = 0.68 (95% CI 0.26–1.11, P = 0.0004, FDR = 0.0039; Figure 2E and Table S5). Shape‐related effects were smaller: Δ solidity mean showed g = 0.49 (95% CI 0.07–0.90, P = 0.0125, FDR = 0.0748), whereas Δ solidity CV showed g = −0.42 (95% CI −0.831 to −0.004, P = 0.0363, FDR = 0.1149). The pair‐level distributions are shown in Figure 2F–I. The T0‐to‐T1 trajectories showed the same pattern. Mean equivalent nuclear diameter and mean nuclear area decreased on average in non‐progression pairs but increased in progression pairs in Center 2 (Figure 2J,K).

**FIGURE 2:**
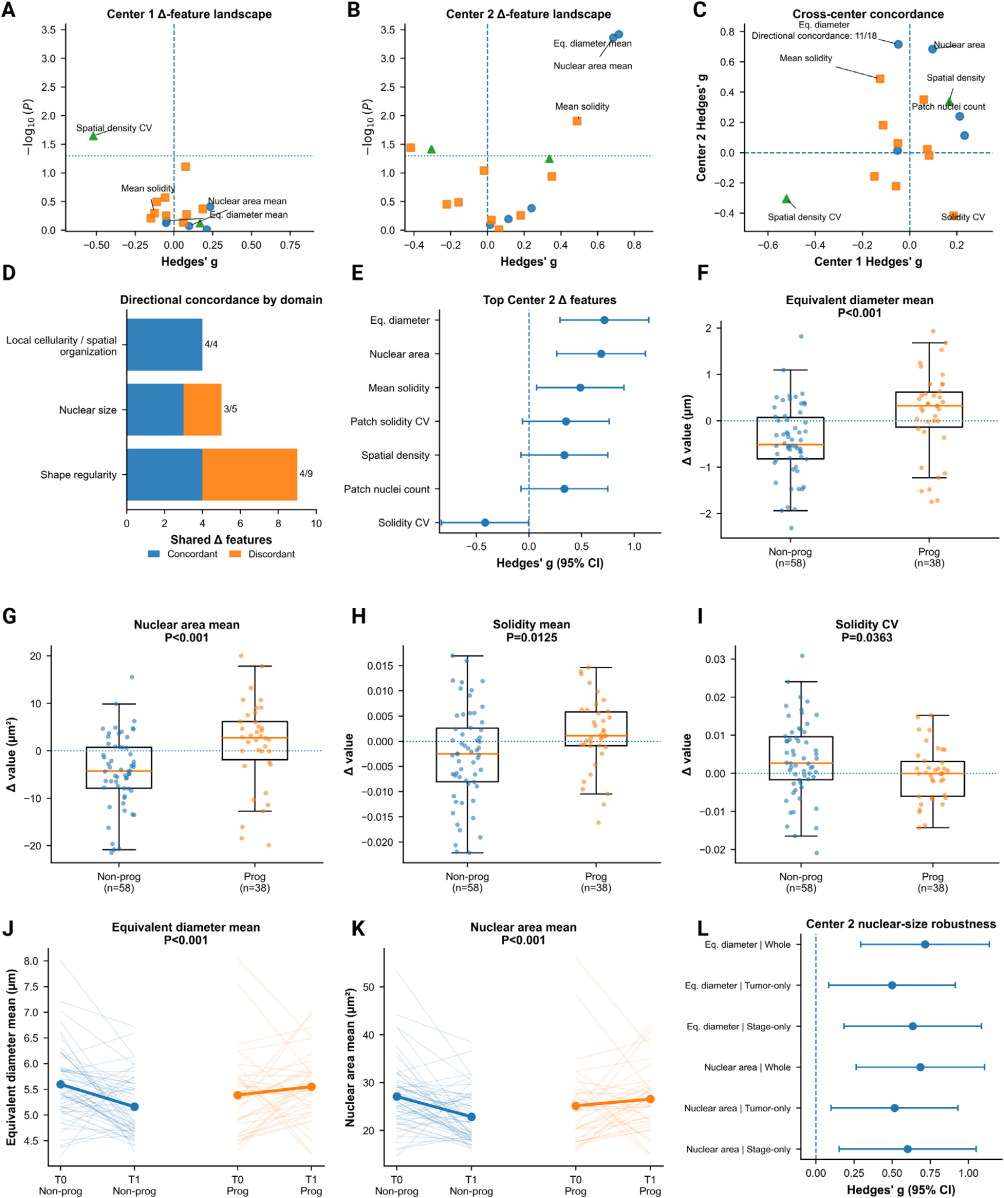
Longitudinal nuclear‐feature changes associated with progression across the two paired cohorts. (A, B) Effect‐size landscapes of the 18 shared pair‐level Δ features in Center 1 and Center 2, respectively. The *x*‐axis shows Hedges’ g for progression vs. non‐progression, and the *y*‐axis shows −log10(P) from the Mann–Whitney *U* test. Colors and symbols denote nuclear size, shape regularity, and local cellularity/spatial organization. (C) Cross‐center comparison of Hedges’ g for the same 18 shared Δ features. Eleven of 18 features showed the same direction of effect in the two centers. (D) Directional concordance summarized by feature domain. (E) Effect sizes and 95% confidence intervals for the leading progression‐associated Δ features in Center 2. (F–I) Patient‐pair distributions of Δ mean equivalent nuclear diameter, Δ mean nuclear area, Δ mean solidity, and Δ solidity coefficient of variation, respectively, in progression and non‐progression groups in Center 2. (J, K) Paired T0‐to‐T1 trajectories for mean equivalent nuclear diameter and mean nuclear area in Center 2. Thin lines represent individual patient pairs and thick lines connect group means. (L) Sensitivity analysis of the two principal nuclear‐size features in Center 2 across the primary whole‐tissue analysis, the pathologist‐confirmed tumor‐only analysis, and the strict stage‐only progression definition. Points indicate Hedges’ g and horizontal bars indicate 95% confidence intervals. Δ denotes T1 minus T0. P values shown in panels A, B, and F–K were calculated using two‐sided Mann–Whitney *U* tests on pair‐level Δ features.

To assess whether the nuclear‐size signal could be explained by differences in non‐tumor tissue composition, we repeated the analysis within pathologist‐annotated tumor‐enriched regions. In Center 2, the progression‐associated direction was preserved after tumor restriction: Δ nuclear area mean showed Hedges' g = 0.52 (95% CI 0.10–0.93; P = 0.013), and Δ equivalent nuclear diameter mean showed Hedges' g = 0.50 (95% CI 0.08–0.91; P = 0.011; Figure 2L). The corresponding effects were weaker and not statistically significant in Center 1 (Δ nuclear area mean: g = 0.25, 95% CI −0.18–0.69, P = 0.414; Δ equivalent nuclear diameter mean: g = 0.09, 95% CI −0.34–0.52, P = 0.568). Thus, tumor restriction preserved the confirmation‐cohort nuclear‐size signal but did not uniformly strengthen the effect across cohorts (Figure S3C and Table S6).

The nuclear‐size findings were also stable across segmentation settings. In the Center 2 cohort, changing the nominal Cellpose diameter from 12 to 18 pixels and the minimum nuclear‐area threshold from 5 to 10 µm^2^ did not change the direction of any of the four nuclear‐size readouts across the 20 alternative parameter–feature comparisons. We also compared Cellpose output with manually reviewed nuclear masks in 12 tumor‐region windows. Pixel‐level precision, recall, Dice, and IoU were 0.620, 0.546, 0.581, and 0.409 in Center 1 and 0.846, 0.606, 0.706, and 0.546 in Center 2, respectively. Removing the border‐clearing step did not change the number of retained predicted nuclei in these validation regions (no‐border/border ratio = 1.00 in both centers; Table S9).

We also examined whether fallback scale assignment affected the nuclear‐size results. After the final slide‐level audit and Center 1 VSI reconstruction, 33 of 364 longitudinal WSIs still required the predefined 0.25 µm/pixel value. All 33 came from Center 1, whereas none of the 192 Center 2 WSIs required fallback scaling. These slides included 20 T0 and 13 T1 specimens and involved 30 of 86 Center 1 pairs. Fallback‐containing pairs occurred at similar frequencies in progression and non‐progression cases (13/35 vs. 17/51; Fisher's exact P = 0.819), and there was no significant imbalance between T0 and T1 (P = 0.248). After excluding all 30 affected Center 1 pairs, 56 pairs remained (22 progression and 34 non‐progression). The whole‐tissue nuclear‐size differences remained small and non‐significant (Δ nuclear area mean: Hedges' g = 0.08, 95% CI −0.46–0.61, P = 0.960; Δ equivalent nuclear diameter mean: g = 0.04, 95% CI −0.49–0.58, P = 0.927). Center 2 contained no fallback‐scaled slides, so this exclusion did not alter its results. The nuclear‐size associations remained significant for Δ nuclear area mean (g = 0.68, 95% CI 0.26–1.11, P = 0.0004) and Δ equivalent nuclear diameter mean (g = 0.72, 95% CI 0.29–1.14, P = 0.0004; Table S10).

Because grade upgrading may overlap with nuclear atypia, we further examined the two principal nuclear‐size features under alternative progression definitions in Center 2. The stage‐only analysis in Center 2 included 30 progression and 58 non‐progression pairs. Nuclear size‐related drift remained directionally consistent and statistically significant, including Δ equivalent nuclear diameter mean (Hedges’ g = 0.63, 95% CI 0.18–1.09, P = 0.003) and Δ nuclear area mean (g = 0.60, 95% CI 0.15–1.05, P = 0.003). Positive effects were also observed for NMIBC‐to‐MIBC transition (10 progression and 58 non‐progression pairs; Δ equivalent nuclear diameter mean: g = 0.86, 95% CI 0.17–1.55; Δ nuclear area mean: g = 0.86, 95% CI 0.18–1.55) and grade‐only upgrading (8 progression and 58 non‐progression pairs; g = 1.06, 95% CI 0.30–1.82 for both features; Figure S3D and Table S6). Because these subgroups were small, they were interpreted as sensitivity analyses rather than independent validation. In separate adjusted single‐feature logistic models using the broad progression definition (38 progression and 58 non‐progression pairs), both Δ equivalent nuclear diameter mean and Δ nuclear area mean remained associated with progression after adjustment for age, sex, and baseline muscle‐invasive status (Table S6). These analyses further supported the robustness of the nuclear size‐related signal in the independent confirmation cohort (Table 2).

**TABLE 2:** Summary of key feature‐level progression‐associated longitudinal drift findings.

Cohort	Progression/non‐progression (n)	Feature/metric	Effect size/summary	P value	FDR
Center 1 discovery	35/51	Δ spatial density CV/patch nuclei‐count CV	SMD (Hedges' g) = −0.52 (95% CI −0.96 to −0.08)	0.0225	0.2029
Center 2 confirmation	38/58	Δ equivalent nuclear diameter mean	SMD (Hedges' g) = 0.72 (95% CI 0.29 to 1.14)	0.0004	0.0039
Center 2 confirmation	38/58	Δ nuclear area mean	SMD (Hedges' g) = 0.68 (95% CI 0.26 to 1.11)	0.0004	0.0039
Center 2 confirmation	38/58	Δ solidity mean	SMD (Hedges' g) = 0.49 (95% CI 0.07 to 0.90)	0.0125	0.0748
Center 2 confirmation	38/58	Δ solidity CV	SMD (Hedges' g) = −0.42 (95% CI −0.831 to −0.004)	0.0363	0.1149
Center 1 vs Center 2	86/96 pairs	Directionally concordant shared Δ features	11/18	—	—

**Note**: Values are derived from pair‐level signed Δ features calculated as T1 minus T0. Standardized mean differences are reported as Hedges' g with 95% confidence intervals. P values were calculated using two‐sided Mann–Whitney *U* tests and FDR by Benjamini–Hochberg correction within each center for feature‐level comparisons. Center 1 was treated as the exploratory discovery cohort and Center 2 as the independent confirmation cohort. The outcome‐informed exploratory drift score is not included in this table because it is retained only as a descriptive supplementary analysis.

To examine whether intercurrent treatment, T0–T1 interval, or procedure type could account for the nuclear‐size findings, we performed additional sensitivity analyses in the paired cohorts. The median T0–T1 interval was 16.0 months (IQR 7.25–28.0) in Center 1 and 12.5 months (IQR 7.0–27.0) in Center 2, with no significant difference between progression and non‐progression pairs in either center (P = 0.057 and P = 0.104, respectively). In Center 2, the nuclear‐size signal remained significant after adjustment for T0–T1 interval, age, sex, baseline muscle‐invasive status, T0 intercurrent intravesical‐treatment exposure, and T0 TURBT status (Δ equivalent nuclear diameter mean: adjusted OR per 1‐SD = 2.58, 95% CI 1.50–4.45, P < 0.001; Δ nuclear area mean: adjusted OR per 1‐SD = 2.50, 95% CI 1.45–4.32, P = 0.001). The signal also remained significant in the most restrictive subset of T0 intravesical‐chemotherapy‐treated TURBT‐to‐TURBT pairs (23 progression and 42 non‐progression pairs; diameter: g = 0.59, 95% CI 0.08–1.11, P = 0.021; area: g = 0.54, 95% CI 0.02–1.05, P = 0.025). Corresponding Center 1 adjusted associations remained non‐significant (diameter: OR = 1.12, 95% CI 0.67–1.85, P = 0.671; area: OR = 1.36, 95% CI 0.81–2.26, P = 0.243). Pairs without documented intercurrent treatment were too few for stable inference (Center 1, n = 5; Center 2, n = 6; Table S11).

### 2.3 Spatial Analysis Shows Broader Nuclear‐Area Distribution Shifts in Progression Pairs

We next examined whether nuclear size‐related drift was reflected in the spatial distribution of tissue‐level nuclear features. For each Center 2 WSI, non‐empty 500‐µm spatial bins were summarized by nuclei count and mean nuclear area. Representative progression and non‐progression pairs showed substantial spatial heterogeneity at both T0 and T1 (Figure 3A,B). Because T0 and T1 were separate pathological specimens, we treated them as independent within‐slide spatial distributions and did not match spatial bins by coordinate.

**FIGURE 3:**
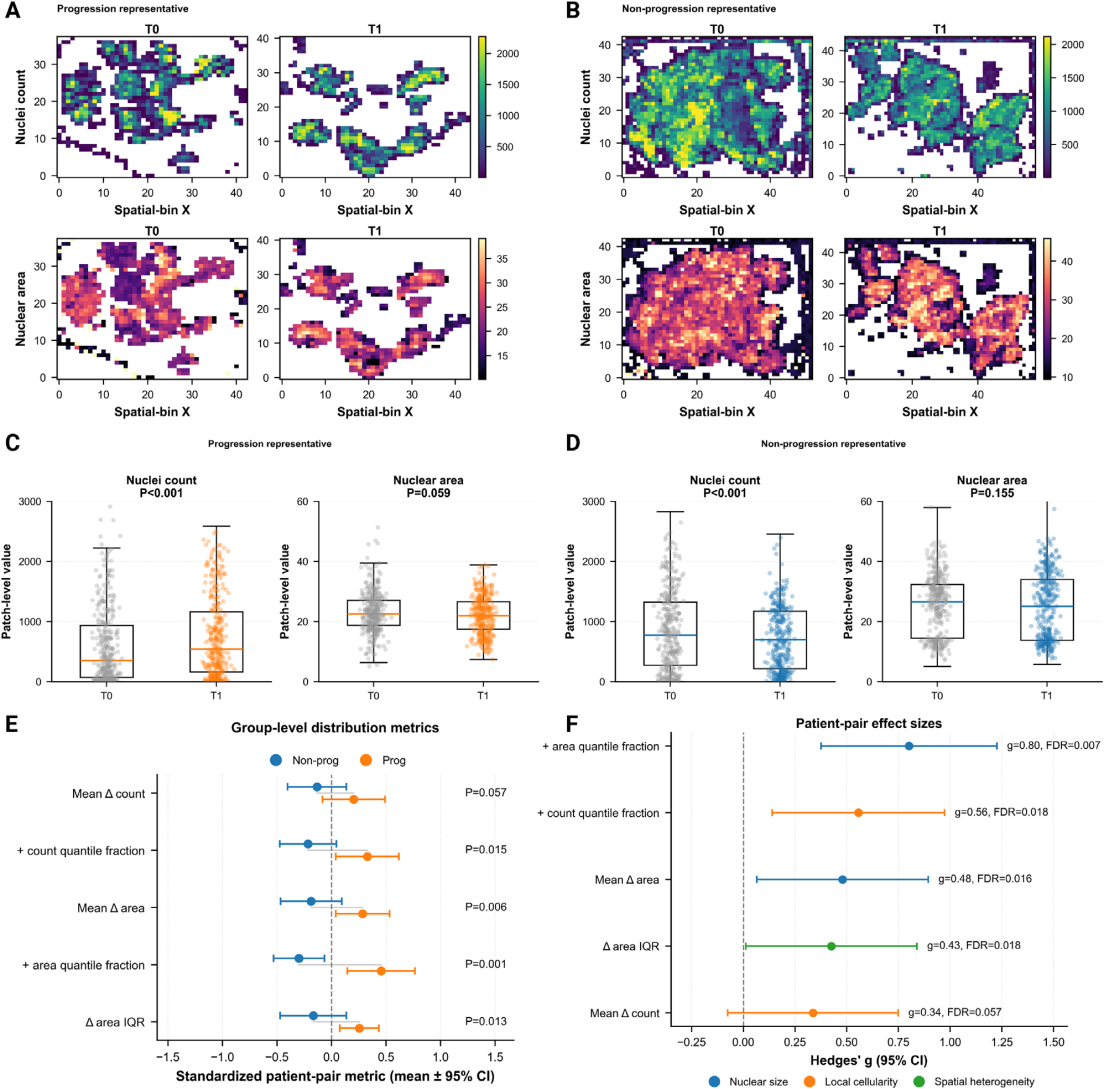
Distribution‐based spatial analysis of longitudinal nuclear change. (A, B) Representative progression and non‐progression pairs showing T0 and T1 spatial‐bin maps of nuclei count and mean nuclear area. (C–D) Spatial‐bin distributions for the same pairs; P values are descriptive Mann–Whitney *U* tests. (E) Standardized patient‐pair distribution metrics in progression and non‐progression groups; points and bars show means and 95% CIs. (F) Hedges’ g with 95% CIs for progression vs. non‐progression; FDR values were Benjamini–Hochberg adjusted. T0 and T1 were analyzed as separate, non‐registered specimens, and cohort‐level inference used the patient pair as the unit of analysis. Positive quantile fraction denotes the proportion of prespecified quantiles with T1 > T0.

The spatial‐bin distributions for the same representative pairs are shown in Figure 3C,D. In the progression example, nuclei‐count distributions differed between T0 and T1 (P < 0.001), whereas the nuclear‐area difference was weaker (P = 0.059). In the non‐progression example, nuclei count also differed between specimens (P < 0.001), whereas nuclear‐area distributions were similar (P = 0.155). These comparisons were descriptive only and were not used as patient‐level evidence.

At the cohort level, we summarized each T0 and T1 spatial distribution before calculating five prespecified metrics at the patient‐pair level (Figure 3E,F). The largest difference involved the positive nuclear‐area quantile fraction (Hedges’ g = 0.80, 95% CI 0.38–1.23, P = 0.0014, FDR = 0.0070). Positive nuclei‐count quantile fraction (g = 0.56, 95% CI 0.14–0.97, P = 0.0147, FDR = 0.0183), mean nuclear‐area shift (g = 0.48, 95% CI 0.06–0.89, P = 0.0063, FDR = 0.0158), and nuclear‐area IQR shift (g = 0.43, 95% CI 0.01–0.84, P = 0.0133, FDR = 0.0183) also differed between groups. Mean nuclei‐count shift was weaker (g = 0.34, 95% CI −0.08 to 0.75, P = 0.0566, FDR = 0.0566). These results indicate that progression was associated with a broader upward shift in the spatial distribution of nuclear area without requiring anatomical correspondence between T0 and T1.

### 2.4 Static WSI Models Provide Contextual Benchmarks

We next used the four‐center institutional WSI resource as a cross‐sectional contextual benchmark for conventional single‐time‐point risk modeling rather than as evidence for longitudinal drift. In Center 1, repeated internal cross‐validation yielded AUCs of 0.72 (95% CI 0.67–0.77), 0.69 (0.65–0.74), and 0.75 (0.71–0.79) for the clinical‐only, UNI2 pathology‐only, and UNI2 fusion models, respectively (Figure 4A and Table 3).

**FIGURE 4:**
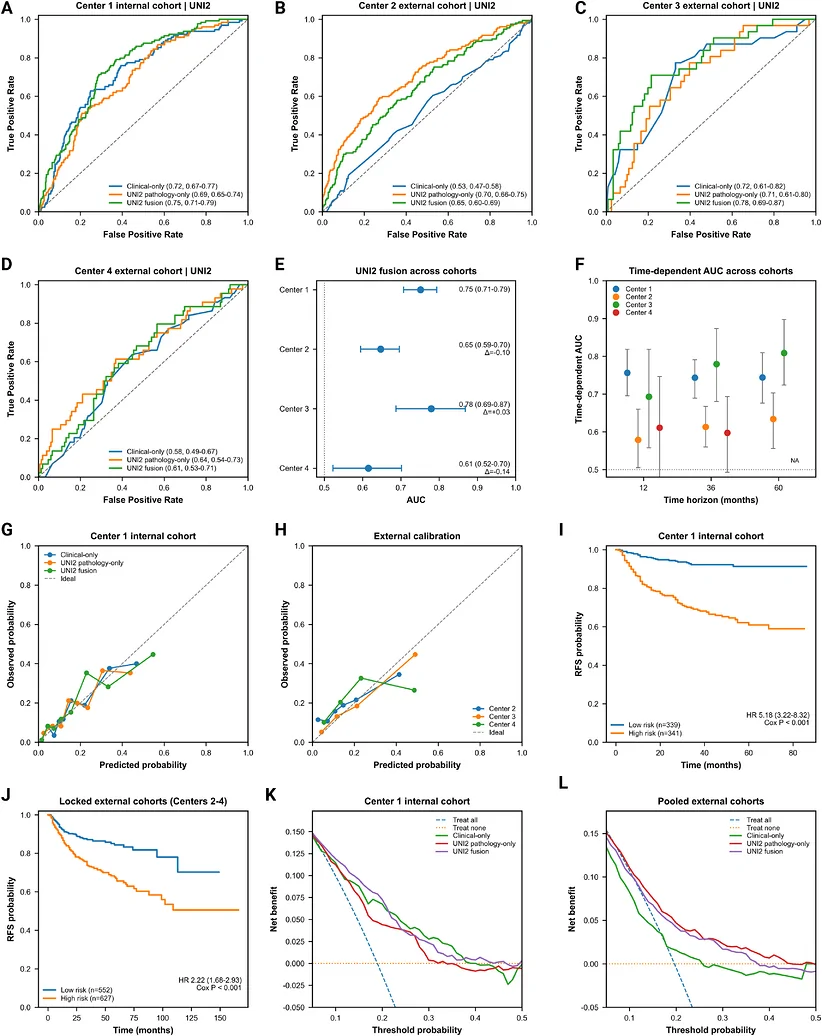
Static WSI contextual benchmarks and locked cross‐center transferability. (A) Internal ROC curves for clinical‐only, UNI2 pathology‐only, and UNI2 fusion models in Center 1. (B–D) Locked external ROC validation in Centers 2–4. (E) Cross‐center UNI2 fusion AUCs. (F) Time‐dependent AUCs at 12, 36, and 60 months. (G,H) Internal and external calibration. (I) Center 1 internal RFS stratification using the median cross‐validated UNI2 fusion score. (J) Pooled locked external RFS stratification using the fixed Center 1‐derived cutoff. (K,L) Internal and pooled external decision curve analysis. Error bars indicate 95% confidence intervals where applicable. Complete backbone comparisons are shown in Figure S4.

**TABLE 3:** Performance and locked external transferability of UNI2‐based WSI contextual baseline models.

Validation setting	Validation cohort	Model	AUC (95% CI)	Δ vs clinical	External Δ vs Center 1 fusion
Internal validation	Center 1	Clinical‐only	0.72 (0.67–0.77)	—	—
Internal validation	Center 1	UNI2 pathology‐only	0.69 (0.65–0.74)	−0.03	—
Internal validation	Center 1	UNI2 fusion	0.75 (0.71–0.79)	+0.03	—
Locked external validation	Center 2	Clinical‐only	0.53 (0.47–0.58)	—	—
Locked external validation	Center 2	UNI2 pathology‐only	0.70 (0.66–0.75)	+0.17	—
Locked external validation	Center 2	UNI2 fusion	0.65 (0.60–0.69)	+0.12	−0.10
Locked external validation	Center 3	Clinical‐only	0.72 (0.61–0.82)	—	—
Locked external validation	Center 3	UNI2 pathology‐only	0.71 (0.61–0.80)	−0.01	—
Locked external validation	Center 3	UNI2 fusion	0.78 (0.69–0.87)	+0.06	+0.03
Locked external validation	Center 4	Clinical‐only	0.58 (0.49–0.67)	—	—
Locked external validation	Center 4	UNI2 pathology‐only	0.64 (0.54–0.73)	+0.06	—
Locked external validation	Center 4	UNI2 fusion	0.61 (0.53–0.71)	+0.03	−0.14

Note: Values are AUCs with 95% confidence intervals. Center 1 internal performance was estimated using repeated stratified cross‐validation. Locked external validation applied the Center 1‐derived preprocessing and fitted models directly to Centers 2–4 without center‐specific retraining or recalibration. Δ vs clinical denotes the model AUC minus the clinical‐only AUC within the same validation cohort; external Δ is shown for UNI2 fusion and denotes the external fusion AUC minus the Center 1 internal fusion AUC. AUC, area under the receiver operating characteristic curve; CI, confidence interval; WSI, whole‐slide image.

When the final Center 1 models were applied unchanged to the external cohorts, UNI2 fusion AUCs were 0.65 in Center 2, 0.78 in Center 3, and 0.61 in Center 4 (Figure 4B–E and Table 3). Thus, transferability varied substantially across institutions. Time‐dependent discrimination showed a similar pattern across follow‐up horizons (Figure 4F and Figure S5A,C), while calibration also varied across external centers (Figure 4G,H). UNI2 fusion scores stratified RFS in Center 1 (HR = 5.18, 95% CI 3.22–8.32; P < 0.001) and in the pooled locked external cohorts (HR = 2.22, 95% CI 1.68–2.93; P < 0.001; Figure 4I,J). Decision curve analysis is shown in Figure 4K,L. No single encoder uniformly outperformed the others across all cohorts; complete comparisons of UNI2, CTransPath, ResNet50, and DenseNet121 are provided in Figure S4 and Table S7. These analyses therefore support the presence of static WSI‐level risk information while also demonstrating heterogeneous cross‐center transportability.

### 2.5 TCGA Anchors Morphology‐Domain Axes to Immune Programs

We then examined TCGA‐BLCA as an orthogonal molecular anchoring layer. Because TCGA‐BLCA does not contain true paired T0/T1 H&E specimens, this analysis did not test longitudinal drift directly. It instead asked whether cross‐sectional morphology‐domain axes, constructed independently from TCGA H&E‐derived features, were associated with transcriptomic programs related to tumor biology. The corresponding pathology‐derived summary axes were constructed from prespecified standardized feature groups, with direction alignment where applicable, as detailed in the Section 4 and Table S4.

The nuclear size axis showed negative correlations with several immune‐active signatures, including CD8 T cells, cytolytic activity, exhaustion checkpoint, T cell‐inflamed signatures, NK cells, IFN‐γ response, and antigen presentation (Figure 5A and Table S8). Hallmark pathway analysis showed a similar pattern, with associations involving immune and inflammatory programs, including interferon response and allograft rejection‐related signatures (Figure 5B).

**FIGURE 5:**
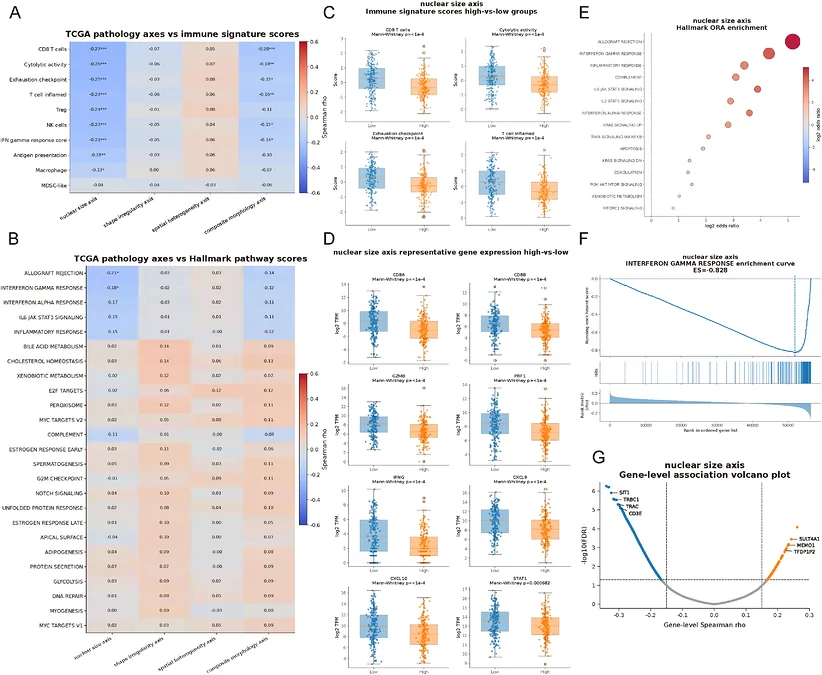
TCGA molecular anchoring of pathology‐derived axes. (A) Correlations between pathology‐derived axes and immune signature scores in TCGA‐BLCA. (B) Correlations between pathology‐derived axes and Hallmark pathway scores. (C) Immune signature scores stratified by high vs. low nuclear size axis groups. (D) Representative immune‐related gene expression differences between high and low nuclear size axis groups. (E) Hallmark over‐representation analysis of nuclear size axis‐associated genes. (F) Enrichment curve for the interferon‐gamma response pathway along the nuclear size axis‐associated gene ranking. (G) Gene‐level association volcano plot for the nuclear size axis. Correlations were assessed using Spearman analysis. TCGA analyses were used for molecular anchoring rather than longitudinal validation.

High‐vs.‐low nuclear size axis comparisons further showed lower immune signature scores and lower expression of representative immune‐related genes in cases with higher nuclear size axis values (Figure 5C,D). Gene‐level and pathway enrichment analyses of nuclear size axis‐associated genes again pointed to immune‐inflammatory and interferon‐related programs (Figure 5E–G and Figure S6). These results do not establish a mechanism, but they provide molecular context for the nuclear size‐related morphology axis and link it to broader immune and interferon‐related tumor states.

### 2.6 Ki‐67 Provides Tissue‐Level Support for Nuclear Size‐Related Drift

We examined Ki‐67 immunohistochemistry in the available IHC subset, focusing on Center 2 where the nuclear size‐related signal was strongest. Representative T1 Ki‐67 fields selected from cases with high and low H&E‐derived nuclear size‐related drift showed visibly different staining patterns (Figure 6A). These images were used for visualization only.

**FIGURE 6:**
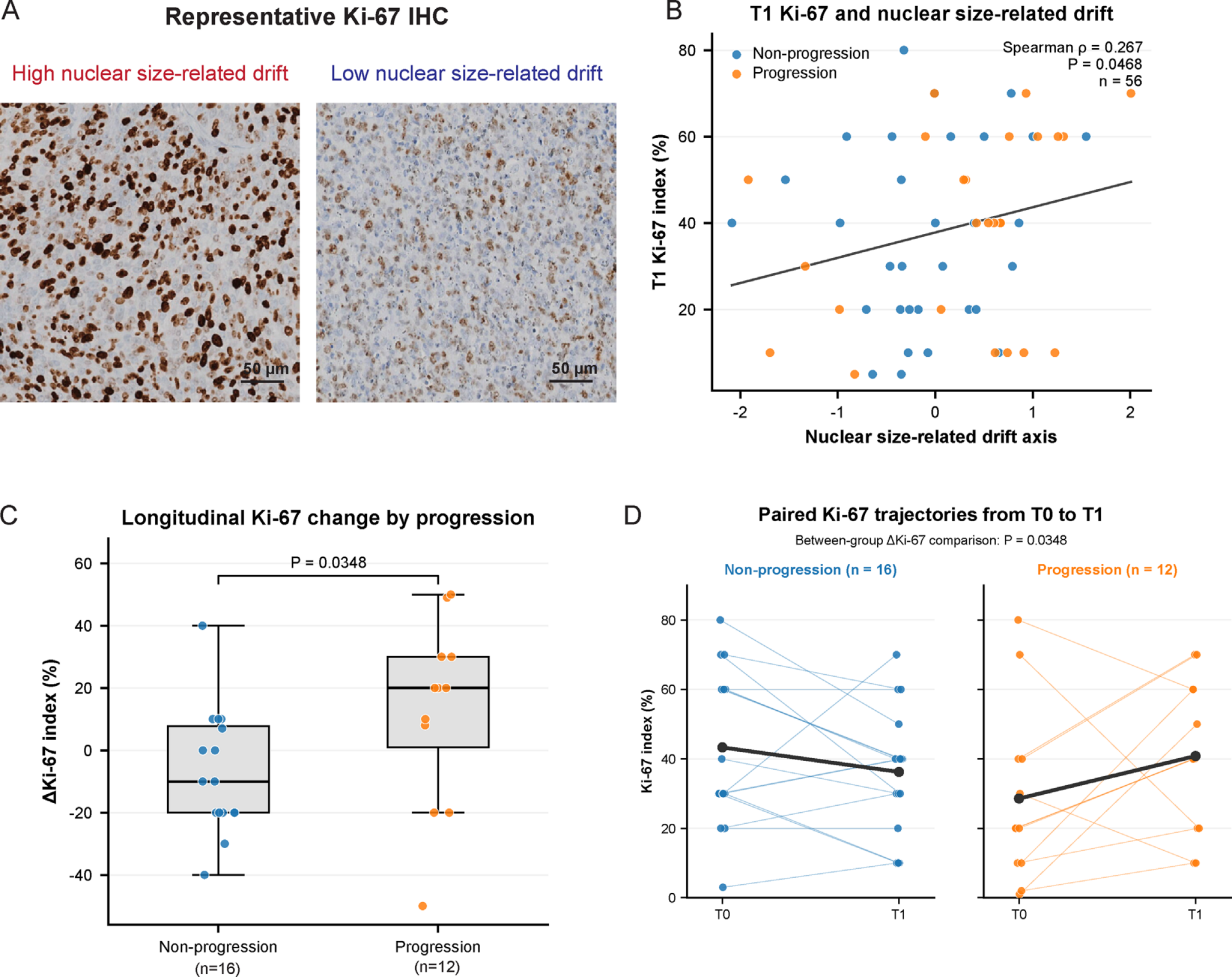
Tissue‐level Ki‐67 anchoring of nuclear size‐related drift. (A) Representative Ki‐67 immunohistochemistry images from Center 2 cases with high and low H&E‐derived nuclear size‐related drift. Scale bars: 50 µm. (B) Association between the T1 Ki‐67 index and the nuclear size‐related drift axis in the Center 2 IHC subset. A fitted line is shown for visualization. Spearman ρ = 0.267, *P* = 0.0468, n = 56. (C) Comparison of ΔKi‐67 between non‐progression and progression groups. ΔKi‐67 was defined as T1 Ki‐67 index minus T0 Ki‐67 index. Mann–Whitney *U* test, *P* = 0.0348. (D) Paired Ki‐67 trajectories from T0 to T1 stratified by progression status. Thin lines indicate individual cases; thick black line indicates the group mean trajectory.

Quantitatively, the T1 Ki‐67 index showed a modest positive correlation with the nuclear size‐related drift axis (Spearman ρ = 0.267, P = 0.0468, n = 56; Figure 6B). Among cases with Ki‐67 measurements at both time points, ΔKi‐67 was higher in progression pairs than in non‐progression pairs (progression n = 12; non‐progression n = 16; P = 0.0348; Figure 6C). Paired trajectories showed a decreasing mean Ki‐67 pattern in non‐progression pairs and an increasing mean pattern in progression pairs (Figure 6D). Because the IHC subset was limited, these findings were interpreted as tissue‐level support rather than independent validation. Together with the TCGA analysis, these IHC findings provided biological anchoring for nuclear size‐related drift.

## 3 Discussion

This study shows that routinely acquired serial H&E specimens can be used to measure direction‐preserving intrapatient morphological change in bladder cancer. Across the two paired cohorts, several nuclear features showed the same direction of association with progression. Nuclear size‐related change was strongest in the independent confirmation cohort, whereas the corresponding effects were weaker in Center 1. Importantly, the nuclear size signal remained evident under a stricter stage‐only progression definition and showed the same positive direction in the NMIBC‐to‐MIBC transition subgroup, reducing the likelihood that the overall association was driven solely by grade relabeling. The Center 2 nuclear‐size associations also persisted after adjustment for T0–T1 interval, T0 intravesical‐treatment exposure, and T0 TURBT status, and in treatment/procedure‐comparable subsets. Because the NMIBC‐to‐MIBC and grade‐only subgroups were small, these estimates were interpreted as sensitivity analyses rather than independent validation. Because the exploratory drift score was assembled after outcome‐informed feature screening and direction alignment, we did not treat its within‐cohort separation as confirmatory evidence. We instead based the biomarker interpretation on individual signed Δ features, the direction of shared feature effects across centers, and the nuclear‐size sensitivity analyses.

The main value of this work is not that H&E slides contain risk‐related information; many cross‐sectional computational pathology studies have already shown that they do [8, 9, 10]. The more specific point is that serial pathology contains temporal information that is usually discarded. In routine practice, T0 and T1 specimens are often interpreted as separate diagnostic events. By organizing them as a within‐patient pair and analyzing signed T1−T0 features, this framework converts repeated pathological sampling into a quantitative readout of tumor evolution [1, 2]. This distinction matters because a tumor that appears aggressive at one time point is not necessarily equivalent to a tumor that is actively shifting toward a more aggressive state.

Nuclear size‐related drift is also a defensible readout because it is both interpretable and biologically plausible. Nuclear enlargement is familiar to pathologists and closely related to conventional assessment of urothelial atypia [16, 17]. Quantitative nuclear morphology and tissue‐architecture features have also been explored in bladder cancer digital pathology and prognostic pathology studies [10, 18]. However, the relevant observation here is longitudinal rather than cross‐sectional. Progression pairs did not merely have larger nuclei at a single time point; they showed a direction‐preserving increase from T0 to T1. The spatial analysis strengthened this interpretation. Progression was associated not only with higher average nuclear‐area change, but also with a broader upward shift across the spatial distribution of nuclear area. This suggests that the signal reflects a wider tissue‐level shift rather than a small number of local outlier regions.

Importantly, the term “nuclear size‐related drift” in this study refers to longitudinal change in two‐dimensional nuclear profiles measured on routine H&E sections rather than direct measurement of three‐dimensional nuclear volume. Apparent nuclear area and area‐derived diameter depend on the plane through which a nucleus is sectioned, and T0 and T1 were obtained from independently sampled pathological specimens. The observed shift may therefore reflect a combination of true biological nuclear enlargement, clonal selection, regional tumor heterogeneity, and sampling variation. We interpret this progression‐associated increase as a population‐level histomorphological readout rather than evidence that individual nuclei physically enlarged in three dimensions between T0 and T1.

The partial mismatch between Center 1 and Center 2 deserves a practical interpretation. Multicenter pathology data are affected by sampling site, tumor content, staining, scanning, specimen type, clinical interval, and treatment history [13, 14]. Under these conditions, exact feature‐level replication is difficult to expect, especially in modestly sized paired cohorts. Center 1 showed a broader pattern of progression‐associated longitudinal drift, whereas nuclear size‐related changes were more pronounced in Center 2. The two cohorts therefore showed directional agreement across several nuclear features, but the magnitude of the nuclear‐size effect differed between centers.

Recent pathology foundation models have improved static WSI representation learning across tumor types and computational pathology tasks [20, 21, 23]. CTransPath and related self‐supervised encoders have also been widely used as histopathology feature extractors [22]. In our contextual analysis, the UNI2 fusion model achieved an internal AUC of 0.75 in Center 1, whereas locked external AUCs were 0.65, 0.78, and 0.61 in Centers 2–4, respectively. No encoder consistently performed best across all cohorts. These findings show that static WSI models contain risk‐related information, but their performance varies across institutions. The relatively stronger transfer observed in Center 3 should not be interpreted as evidence of intrinsic biological similarity to the development cohort. Because no center‐specific retraining or recalibration was performed, the observed differences likely reflect the combined effects of institutional case mix, tissue sampling and processing, staining characteristics, scanner‐related variation, and other site‐specific factors. The present data do not allow these contributions to be disentangled individually, and we therefore regard the higher Center 3 performance as an empirical feature of cross‐center transportability rather than evidence for a specific causal explanation. This static analysis addresses a different question from the paired longitudinal framework and was therefore retained as a contextual benchmark rather than evidence for longitudinal drift.

The biological anchoring analyses helped place the nuclear size‐related morphology domain in context. Ki‐67 provided tissue‐level support, with a modest positive association between T1 Ki‐67 and the nuclear size‐related drift axis and higher ΔKi‐67 in progression pairs [27, 28]. TCGA‐BLCA provided an orthogonal transcriptomic layer for molecular anchoring [24, 25]. The immune and interferon‐related interpretation was further placed in context by pan‐cancer immune‐landscape work [26]. These findings should be interpreted carefully. The IHC subset was limited, and TCGA does not contain true paired T0/T1 H&E specimens. Thus, these analyses do not prove mechanism and do not independently validate longitudinal drift. Their role is narrower but still useful: they suggest that the morphology‐domain signal is connected to broader tumor biology rather than being only a numerical image feature.

Several limitations remain. First, this was a retrospective study, and T0/T1 specimens were not collected under a standardized prospective sampling protocol. Differences in tumor region, tissue amount, specimen type, clinical interval, and treatment exposure may influence measured drift. The pathologist‐annotated tumor‐region sensitivity analysis reduced contamination from large non‐tumor components and preserved the nuclear‐size signal in Center 2; however, the weaker Center 1 effects and residual non‐tumor elements within tumor‐enriched regions indicate that sampling heterogeneity cannot be fully eliminated. Second, T0 and T1 slides came from separate pathological specimens rather than registered serial sections. Accordingly, nuclear area and equivalent diameter represent two‐dimensional section profiles and are influenced by sectioning plane and specimen sampling; they should not be interpreted as direct measurements of three‐dimensional nuclear volume. Because routine H&E sections capture random two‐dimensional nuclear profiles, smaller profiles may arise from peripheral sectioning through otherwise larger nuclei. The minimum‐area sensitivity analysis showed that the progression‐associated direction was not dependent on a single small‐object exclusion threshold, but it cannot eliminate the intrinsic influence of the sectioning plane on two‐dimensional nuclear‐size distributions. The spatial maps therefore summarize within‐slide feature distributions and should not be interpreted as direct region‐to‐region tissue registration. Third, nuclear segmentation and size conversion depend on tissue quality, staining, scanner metadata, and microns‐per‐pixel calibration. QC‐enriched analyses and default‐scale flagging addressed part of this issue, but residual technical variation cannot be fully excluded. Fourth, the exploratory drift score was outcome‐informed and is therefore susceptible to optimistic bias. We retained it only as a descriptive supplementary analysis and did not use it as evidence for biomarker validity or clinical prediction. Finally, Ki‐67 and TCGA analyses supported biological interpretation but did not provide mechanistic proof or independent longitudinal validation.

Intercurrent treatment and procedure differences represent an additional source of potential confounding. Although the Center 2 nuclear‐size signal remained significant after adjustment for T0–T1 interval, T0 intravesical‐treatment exposure, and T0 TURBT status and in treatment/procedure‐comparable subsets, the no‐intercurrent‐treatment subsets were very small (Center 1, n = 5; Center 2, n = 6). Treatment exposure was represented by recorded postoperative treatment categories rather than detailed dose, duration, or treatment‐response data; residual treatment‐related confounding therefore cannot be excluded.

In conclusion, this study identifies progression‐associated longitudinal nuclear drift in bladder cancer, with nuclear size‐related change most clearly observed in the independent confirmation cohort. More broadly, it shows that serial pathology material collected during routine care can be reorganized into signed digital readouts of intrapatient tumor evolution. This moves digital pathology beyond static slide‐level risk modeling and provides a practical basis for studying how tumors change within patients over time. Prospective cohorts with standardized sampling, treatment annotation, and locked biomarker definitions will be needed before clinical deployment.

## 4 Experimental Section

### 4.1 Study Design and Cohort Organization

This multicenter retrospective study examined whether serial H&E whole‐slide images could capture directional intrapatient morphological change in bladder cancer. Institutional cohorts were collected from four hospitals: Xuzhou Central Hospital Affiliated to Medical School of Southeast University (XZCH, Center 1), The Affiliated Linyi People's Hospital of Shandong Second Medical University (LYPH, Center 2), The Affiliated Suqian Hospital of Xuzhou Medical University (SQH, Center 3), and The Affiliated Huaian No.1 People's Hospital of Nanjing Medical University (HNH, Center 4). Public TCGA‐BLCA data were used for transcriptomic anchoring.

The institutional data were organized into two complementary analytical layers. The primary longitudinal analysis focused on paired H&E whole‐slide images from patients with eligible serial pathological specimens in Centers 1 and 2. For each patient, the earlier pathological specimen was labeled as T0 and the later specimen as T1. Center 1 served as the discovery cohort, and Center 2 served as the independent confirmation cohort. After longitudinal eligibility assessment, pair linkage, and quality control, Center 1 retained 86 unique patients contributing 86 valid T0–T1 pairs, whereas Center 2 retained 96 unique patients contributing 96 valid pairs. Each patient contributed only one T0–T1 pair to the final longitudinal analysis.

In parallel, we assembled 1977 institutional H&E whole‐slide images from 1660 patients across Centers 1–4 as a static digital pathology resource for foundation‐model contextual analysis. This broader cross‐sectional resource represented a separate analytical layer and was not the direct denominator of the paired longitudinal analysis. These cross‐sectional slides were not used to define longitudinal drift. TCGA‐BLCA was not treated as longitudinal validation because true paired T0/T1 H&E specimens were not available. Cohort characteristics and analytic sample sizes are summarized in Table 1 and Tables S1–S3. Ethical approval and consent waiver are described in the Ethics Statement.

### 4.2 Definition of Paired Time Points, Progression, and Pair Eligibility

For each longitudinal pair, T0 referred to the earlier pathological specimen and T1 to the later specimen obtained during follow‐up, recurrence evaluation, resection, or subsequent clinical management. Progression status was assigned at the pair level using the documented pair‐level pathological progression annotation, reflecting pathological worsening from T0 to T1. Stage upgrading, NMIBC‐to‐MIBC transition, and documented grade upgrading were summarized as component patterns and were examined in sensitivity or subtype analyses; the broad progression label was not reconstructed solely from these derived fields. Recurrence alone was not classified as progression; recurrent pairs without documented pathological worsening were classified as non‐progression.

Pairs were retained only when patient identity, time‐point assignment, WSI linkage, progression status, and extracted pathology features were unambiguous. Pairs with uncertain patient linkage, inconsistent progression annotation, invalid WSI‐derived features, or incomplete feature tables were excluded. Because grade upgrading may partly overlap with nuclear atypia, we applied two stricter derived definitions in sensitivity analyses. Strict stage‐or‐grade progression required a T0‐to‐T1 increase in pathological stage or grade in the standardized paired fields, whereas strict stage‐only progression required pathological stage worsening; grade‐only upgrading did not count under the stage‐only definition. For additional subtype sensitivity analyses, NMIBC‐to‐MIBC transition was evaluated separately, and grade‐only upgrading was defined operationally as broad progression without pathological stage progression. The broad non‐progression group was used as the common reference for all subtype comparisons.

For the treatment/interval sensitivity analysis, the recorded T0 recurrence‐free interval was used as the T0–T1 interval because it represented the time from the T0 operation/specimen to the recurrence/resection generating T1. T0 postoperative treatment was treated as the documented intercurrent exposure after T0 and before T1. Accordingly, for pairs with documented treatment, the T0 specimen preceded, and the T1 specimen followed the intercurrent treatment; pairs with no documented intercurrent treatment were coded separately. Recorded T0 and T1 surgical procedures were reported as surgery/procedure type. T1 postoperative treatment occurred after acquisition of the T1 specimen and was not used as a pre‐T1 exposure covariate.

### 4.3 Pair‐Level Δ Features and Longitudinal Digital Readouts

Pair‐level Δ features were used as the primary longitudinal digital readouts. For each feature, Δ was calculated as the T1 value minus the corresponding T0 value. This signed formulation preserved the direction of intrapatient morphological change, rather than measuring only the magnitude of difference between two specimens.

The final biomarker interpretation focused on nuclear size‐related drift, represented mainly by Δ equivalent nuclear diameter mean and Δ nuclear area mean. Shape regularity, local cellularity, and spatial organization features were retained to describe the broader longitudinal drift landscape and to assess whether the nuclear size signal occurred within a wider progression‐associated tissue change.

### 4.4 Whole‐Slide Image Processing and Nuclear Feature Extraction

We processed H&E whole‐slide images with a fixed patch‐based nuclear feature extraction workflow using OpenSlide‐compatible WSI reading and tiling procedures [29]. The workflow first identified tissue‐containing regions from low‐resolution slide thumbnails and then extracted high‐resolution tiles from these regions. Near‐background and low‐information areas were removed before nuclear segmentation. Because the primary workflow sampled tissue‐containing regions rather than being restricted to pathologist‐annotated tumor, we additionally performed a dedicated tumor‐region‐restricted sensitivity analysis. Tumor‐containing/tumor‐enriched regions were delineated on all longitudinal H&E WSIs by pathological review using a standardized QuPath protocol, with the aim of encompassing all clearly identifiable and evaluable tumor areas rather than selecting high‐density or high‐grade hotspots. Large areas of benign urothelium, tumor‐free stroma or muscularis, inflammation without identifiable tumor, necrosis, hemorrhage, severe cautery/crush artifact, tissue folds, and out‐of‐focus tissue were excluded. Minor stromal, inflammatory, vascular, and fibrovascular‐core components intermingled within otherwise tumor‐enriched regions were retained. Only nuclei whose centroids were located within the annotated tumor ROIs were retained for recomputation of slide‐level features and pair‐level T1−T0 changes.

Nucleus instance segmentation used Cellpose with a fixed nominal nuclear diameter of 15 pixels [30]. Nuclei touching tile borders were removed, and objects with a nuclear area < 5 µm^2^ were excluded. The 5‐µm^2^ cutoff was intended to remove very small segmented objects likely to represent fragments or segmentation artifacts rather than to define a biological lower bound for nuclear size. Robustness to this exclusion rule was examined using a more restrictive 10‐µm^2^ minimum‐area threshold in combination with Cellpose diameters of 12, 15, and 18 pixels. Across the resulting parameter combinations, the direction of all four prespecified nuclear‐size readouts was preserved. We did not re‐include objects below 5 µm^2^ because these objects could not be reliably distinguished from segmentation fragments. We examined the sensitivity of the nuclear‐size measurements to these settings by varying the Cellpose diameter (12, 15, and 18 pixels) and minimum nuclear‐area threshold (5 and 10 µm^2^) in the Center 2 longitudinal cohort. Segmentation accuracy was also assessed in 12 manually reviewed tumor‐region windows, with six sampled from each center. Pixel‐level precision, recall, Dice coefficient, and intersection‐over‐union (IoU) were calculated against the reviewed nuclear masks. The effect of border removal was examined in the same validation regions. For each retained nucleus, we measured area, centroid coordinates, major‐axis length, minor‐axis length, perimeter, and solidity [31]. Nuclear area was converted from pixels to µm^2^ as *A*  = *A_px_*  ×*MPP_x_*  ×  *MPP_y_*. Equivalent nuclear diameter was not measured independently but was derived from the segmented two‐dimensional nuclear area as *d_eq_* =  √(4*A*/π), corresponding to the diameter of a circle with the same area as the segmented nuclear profile. Slide‐level nuclear area and equivalent‐diameter means were calculated across all retained nuclei, and the corresponding longitudinal Δ features were defined as the T1 slide‐level mean minus the T0 slide‐level mean. Aspect ratio was calculated as major‐axis length divided by minor‐axis length, and circularity as 4π × area/perimeter^2^.

We converted morphometric measurements to micrometer‐based units using slide‐level MPP information. When metadata review could not recover a reliable native scale, we used a predefined value of 0.25 µm/pixel and flagged the slide as fallback‐scaled. After the final Center 1 VSI reconstruction, we re‐audited scale provenance for all longitudinal WSIs. For the sensitivity analysis, we excluded the entire T0–T1 pair whenever either slide still required fallback scaling and then repeated the four prespecified nuclear‐size comparisons. Key nuclear and derived feature definitions are provided in Table S4. MPP was audited for all longitudinal WSIs, and center‐specific scale distributions are provided in Table S9.

### 4.5 Multi‐Scale Aggregation and Quality Control

We aggregated nucleus‐level outputs at patch, spatial‐bin, and slide levels. For local nuclear‐abundance summaries, retained nuclei were assigned by centroid coordinates to fixed 500 × 500 µm spatial bins. Patch nuclei count was defined as the number of retained nuclei within each non‐empty spatial bin; patch nuclei count mean was the mean of these counts across non‐empty bins, and patch nuclei count CV was their coefficient of variation. These count‐based metrics were not additionally normalized by tissue area and were therefore interpreted as coarse measures of local nuclear abundance rather than formal cell density. All retained segmented nuclei were included without cell‐type classification. Spatial density was reported separately using the same fixed 500 × 500 µm spatial grid. Retained nuclei were assigned to spatial bins according to their physical centroid coordinates, and only non‐empty bins contributed to these summaries. For each non‐empty bin *j*, nuclear density was calculated as *D_j_* = *n_j_*/*A_bin_*, where *n_j_* is the number of retained nuclei and *A_bin_* = 500 µm × 500 µm = 0.25 mm^2^. Spatial density mean was the mean of *D_j_* across non‐empty bins, and spatial density CV was the coefficient of variation of *D_j_*. For the longitudinal feature tables used here, normalization used the fixed full‐bin area rather than the measured tissue‐covered area within each bin. The 1024 × 1024‐pixel tiles used for Cellpose inference were processing units and were distinct from these 500 µm analytical spatial bins. Slide‐level summaries captured nuclear morphology, local nuclear abundance, fixed‐bin‐area spatial density, and spatial heterogeneity using the mean, standard deviation, median, and coefficient of variation where appropriate. Within the broader local‐cellularity domain, nuclei‐count features were interpreted specifically as nuclear‐abundance summaries rather than density measures. Before analysis, features were grouped into prespecified interpretable pathology domains: nuclear size, shape regularity, local cellularity, and spatial organization.

Slide‐level feature tables were linked to longitudinal clinical records using patient identifiers, WSI identifiers, and standardized T0/T1 labels. For each valid pair, we calculated signed Δ features as the T1 value minus the T0 value. These pair‐level Δ features formed the basis of the longitudinal analyses.

Quality control considered detected nuclei count, valid tissue‐containing patches or spatial units, image‐scale metadata, and processing warning flags. A pair was flagged when either time point had fewer than 500 detected nuclei or fewer than 5 valid tissue‐containing patches or spatial units. These flags did not automatically remove pairs from the primary cohort; instead, they defined QC‐enriched subsets for sensitivity analyses. Patch‐level and spatial‐bin summaries described the spatial distribution and burden of drift within each pair. The main statistical inference remained at the patient‐pair level, so patches and spatial bins were not treated as independent biological samples.

### 4.6 Discovery, Confirmation, and Spatial Drift Analyses

Center 1 served to identify progression‐associated signed nuclear morphological change, and Center 2 tested whether the same direction of change appeared in an independent paired cohort. All primary comparisons used pair‐level signed Δ features. Cross‐center consistency was evaluated by examining whether shared nuclear features changed in the same direction in both cohorts, without requiring identical effect sizes for individual features across centers.

We also calculated an exploratory drift score as an outcome‐informed descriptive summary in the Center 1 discovery cohort. The candidate pool comprised the 18 shared pair‐level Δ features used in the cross‐center feature analysis. Features were ranked by the absolute value of the Center 1 Hedges' g for progression vs. non‐progression, with the two‐sided Mann–Whitney *U*‐test P value used only as a secondary ordering criterion, and the top 10 features were retained. These were Δ spatial density CV, Δ patch nuclei‐count CV, Δ nuclear area CV, Δ equivalent nuclear diameter CV, Δ solidity CV, Δ spatial density mean, Δ patch nuclei count mean, Δ circularity mean, Δ solidity mean, and Δ aspect ratio mean. Each selected Δ feature was z‐standardized across the valid Center 1 pairs and multiplied by the sign of its Center 1 Hedges' g so that higher aligned values represented the progression‐associated direction. The exploratory drift score for pair i was the equal‐weight mean of the 10 aligned standardized components:

$$Driftscore_{i}=\frac{1}{K}\;\sum_{k\;=1}^{K}s_{k}z_{ik},K=10$$

where z*_ik_* denotes the z‐standardized signed Δ value of feature *k* for pair *i*, s*_k_* denotes the sign of the Center 1 Hedges' g for feature *k*, and *K* = 10 denotes the number of selected features. No additional model fitting, regression weighting, PCA, or dimensionality reduction was used. Because both feature ranking and direction alignment used the Center 1 progression label, the score was outcome‐informed and was retained only as an exploratory descriptive summary rather than independent evidence of association, biomarker validation, or clinical prediction.

For the Center 2 spatial analysis, nucleus‐level measurements were aggregated within 500‐µm spatial bins. Each non‐empty bin was summarized by nuclei count and mean nuclear area. T0 and T1 were treated as separate within‐slide spatial distributions; no T0 bin was matched to a T1 bin by coordinate, and no spatial correspondence or anatomical registration between T0 and T1 bins was assumed. Representative maps therefore show the spatial distribution of each feature within each specimen rather than region‐to‐region change between registered tissue locations.

For each patient pair, we derived five distribution‐based metrics: mean nuclei‐count shift; positive nuclei‐count quantile fraction; mean nuclear‐area shift; positive nuclear‐area quantile fraction; and nuclear‐area IQR shift. Nuclear‐area IQR shift was defined as [Q75(T1) − Q25(T1)] − [Q75(T0) − Q25(T0)] for the spatial‐bin mean nuclear‐area distributions. Positive quantile fractions were defined as the fraction of prespecified 10th–90th percentile levels at which the T1 quantile exceeded the corresponding T0 quantile. Progression and non‐progression groups were compared at the patient‐pair level using two‐sided Mann–Whitney *U* tests. Hedges’ g with 95% confidence intervals and Benjamini–Hochberg FDR across the five metrics were reported. Spatial‐bin tests for the representative pairs were descriptive only.

### 4.7 Static Foundation‐Model WSI Contextual Analysis

Static WSI models were used only as cross‐sectional contextual benchmarks for recurrence risk and were not used to define longitudinal drift. Tissue‐containing patches were resized and normalized using a fixed Vahadane reference template before encoding with UNI2, CTransPath, ResNet50, or DenseNet121 [32]. Patch embeddings were aggregated into slide‐level representations. Pathology representations combined PCA‐reduced mean, standard‐deviation, and top‐decile feature blocks with global, pairwise, and spatial heterogeneity summaries. Clinical‐only models used eight prespecified variables: age ≥ 65 years, sex, recurrent presentation, multiple tumors, tumor size > 3 cm, high grade, muscle‐invasive disease, and concomitant CIS.

Center 1 internal performance was estimated using repeated stratified five‐fold cross‐validation with five repeats at the WSI level. Median imputation, standardization, PCA transformation, and logistic‐regression fitting were performed within each training fold, and AUC 95% confidence intervals were derived by bootstrap resampling. The final pipeline was then refitted on all Center 1 WSIs and applied unchanged to Centers 2–4. No target‐center imputation fitting, standardization fitting, PCA fitting, coefficient estimation, cutoff optimization, or recalibration was performed. Stain normalization used a fixed reference template and was not fitted using the external cohorts. For Kaplan–Meier analyses, Center 1 risk groups were defined using the median cross‐validated UNI2 fusion score. Pooled locked external risk groups across Centers 2–4 were defined using the fixed cutoff derived from the final Center 1 UNI2 fusion model. The supplementary Center 2 subgroup analyses used this same fixed Center 1‐derived cutoff; no cutoff was re‐estimated in any external cohort. Time‐dependent discrimination was evaluated at 12, 36, and 60 months, and calibration was assessed using the corresponding model predictions. Decision‐curve analysis was performed using the net‐benefit framework [33]. For WSI‐timepoint RFS analyses, Cox models used robust variance clustered by patient ID.

### 4.8 Biological Anchoring Using TCGA and Immunohistochemistry

We used TCGA‐BLCA and immunohistochemistry as biological anchoring layers rather than as longitudinal validation. Because TCGA‐BLCA does not contain true paired T0/T1 H&E specimens, TCGA analyses examined cross‐sectional morphology‐domain axes related to the longitudinal drift features, not direct T0‐to‐T1 change.

Two distinct types of pathology‐derived summary axes were used and were constructed independently. For the institutional IHC analysis, the nuclear size‐related drift axis was based on pair‐level changes in mean equivalent nuclear diameter and mean nuclear area. Within each center, before endpoint‐specific filtering, each component was standardized as z = (x – μc)/σc using the center‐specific mean and population standard deviation. The nuclear size‐related drift axis was defined as A_NSD = [z(Δ equivalent nuclear diameter mean) + z(Δ nuclear area mean)]/2. The two components therefore received equal weight, and no PCA, outcome‐dependent weighting, or fitted prediction model was used for this axis.

For TCGA‐BLCA, cross‐sectional pathology axes were constructed independently from TCGA H&E‐derived nuclear features using predefined signed robust‐z averages. Robust standardization was defined as zR = [x–median(x)]/[1.4826 × MAD(x)], with ordinary mean/SD z‐standardization used only when MAD was degenerate. The nuclear size axis comprised area mean, area median, equivalent‐diameter mean, equivalent‐diameter median, and patch‐area mean, all in the positive direction. The shape irregularity axis assigned negative directions to mean solidity and mean circularity and positive directions to mean aspect ratio, solidity CV, circularity CV, aspect‐ratio CV, patch‐solidity‐mean CV, patch‐circularity‐mean CV, and patch‐aspect‐mean CV. The spatial heterogeneity axis comprised patch nuclei‐count CV, spatial‐density CV, density CV, tile nuclei‐count CV, tile‐density CV, patch‐area‐mean CV, patch‐solidity‐mean CV, and patch‐circularity‐mean CV, all in the positive direction. Candidate features that were absent, had fewer than 10 non‐missing values, or had fewer than 3 unique values were omitted. Available signed components were equally weighted and averaged, followed by robust standardization of the resulting domain axis. The composite morphology axis was the equal‐weight mean of the nuclear size, shape irregularity, and spatial heterogeneity axes, followed by robust standardization. For patients with multiple TCGA WSIs, slide‐level values were averaged at the patient level. No PCA or fitted coefficients were used in these TCGA pathology‐axis definitions, and no fitted parameters from the institutional longitudinal cohorts were transferred to TCGA.

Transcriptomic activity was summarized using immune signature and pathway scores, including GSVA‐derived pathway activity and MSigDB Hallmark gene sets, and associations with pathology‐derived axes were assessed using Spearman correlation [34, 35]. Cytolytic activity and IFN‐γ‐related immune programs were interpreted using previously described transcriptomic immune‐signature frameworks [36, 37]. Gene‐level associations were further used for over‐representation and enrichment analyses with Hallmark gene sets, with emphasis on immune, inflammatory, proliferation‐related, and tissue‐remodeling programs.

Ki‐67 immunohistochemistry was examined in the available IHC subset, mainly in Center 2, where nuclear size‐related drift showed the clearest feature‐level signal. Representative Ki‐67 images were selected from T1 specimens at the high and low ends of the H&E‐derived nuclear size‐related drift axis for visualization only. Quantitatively, the T1 Ki‐67 index was correlated with the nuclear size‐related drift axis using Spearman analysis. For cases with Ki‐67 measurements at both time points, we calculated ΔKi‐67 = Ki‐67*_T1_* − Ki‐67*_T0_* and compared ΔKi‐67 between progression and non‐progression pairs using the Mann–Whitney *U* test. Paired Ki‐67 trajectories were plotted from T0 to T1 with group mean trajectories overlaid. HER2 analyses were retained as exploratory tissue‐level context.

### 4.9 **S**tatistical Analysis

Continuous variables were summarized as medians with interquartile ranges or means with standard deviations, and categorical variables as counts and percentages. Pair‐level signed Δ features were compared between progression and non‐progression pairs using the Mann–Whitney *U* test. We reported standardized mean differences as Hedges' g with 95% confidence intervals and calculated the confidence intervals using the standard large‐sample variance approximation for Hedges' g. False discovery rate adjustment was applied to feature‐level comparisons within each cohort using the Benjamini–Hochberg procedure [38]. The primary longitudinal analyses used signed pair‐level Δ features, with Center 1 as the discovery cohort and Center 2 as the independent confirmation cohort. Interpretation focused on prespecified pathological domains, especially nuclear size‐related drift, rather than isolated features alone. The exploratory drift score was analyzed as a descriptive summary and was not treated as a locked clinical prediction model.

Sensitivity analyses examined stricter progression definitions, QC‐enriched subsets, and adjusted associations between key nuclear size‐related Δ features and progression. To reduce circularity between grade upgrading and nuclear morphometry, we examined the two principal nuclear‐size features under the primary broad progression definition, strict stage‐or‐grade progression, strict stage‐only progression, NMIBC‐to‐MIBC transition, and grade‐only upgrading definitions, using the broad non‐progression group as the common reference. Key nuclear size‐related features were further examined using single‐feature logistic regression models adjusted for age, sex, and baseline muscle‐invasive disease status. Treatment/interval sensitivity analyses summarized the T0–T1 interval, T0 intercurrent postoperative treatment, and T0/T1 surgery/procedure type by center and progression group. Single‐feature logistic models for the two principal nuclear‐size Δ features were additionally adjusted for T0–T1 interval, T0 intravesical‐treatment exposure, and T0 TURBT status together with age, sex, and baseline muscle‐invasive disease status. Restricted analyses were performed among pairs receiving T0 intravesical chemotherapy, among TURBT‐to‐TURBT pairs, and among pairs meeting both criteria. Pairs without documented intercurrent treatment were summarized separately but were too few for stable effect‐size inference. For foundation‐model analyses, discrimination was evaluated using AUC with 95% confidence intervals. Kaplan–Meier curves were used for visualization, and Cox regression with robust variance clustered by patient ID was used for WSI‐timepoint landmark analyses [39, 40]. FDR‐adjusted *P* < 0.05 was interpreted as feature‐level statistical significance; other prespecified or exploratory analyses used two‐sided *P* < 0.05 unless otherwise stated.

Analyses were performed using Python and R. Nuclear segmentation was performed using Cellpose with a fixed nominal nuclear diameter of 15 pixels. Statistical analyses and visualization were conducted using standard Python and R scientific‐computing packages [41, 42]. Key analytical parameters are described in the relevant Experimental Section subsections and Supporting Information.

## Author Contributions

X. L. and C. H. H. conceived and designed the study. X. L. performed data curation, computational analysis, statistical analysis, figure preparation, and manuscript drafting. Z. D. S. and G. C. W. contributed to cohort construction, clinical data collection, and result interpretation. J. M. W., Y. L., Y. G., X. L., D. W., S. C. Z., H. H., Y. B. S., F. L. M., Y. Z., and Z. G. J. contributed to pathological review, case coordination, or methodological support. C. H. H. supervised the study. All authors reviewed and approved the final manuscript.

## Ethics Statement

This multicenter retrospective study was approved by the Ethics Committee of Xuzhou Central Hospital Affiliated to Medical School of Southeast University (approval no. XZXY‐LK‐20250328‐0037) and the Ethics Committee of the Affiliated Linyi People's Hospital of Shandong Second Medical University (approval no. 202601‐H‐010). The requirement for written informed consent was waived because the study used de‐identified retrospective clinicopathological data and archival pathological specimens.

## Conflicts of Interest

The authors declare no conflicts of interest.

## Supporting information




**Supporting File**: advs78299‐sup‐0001‐SuppMat.docx.

## Data Availability

The data that support the findings of this study are available on request from the corresponding author. The data are not publicly available due to privacy or ethical restrictions.
