# Supplementary material for: Serial H&E Pathology Reveals Progression‐Associated Nuclear Size Drift as a Longitudinal Digital Readout of Bladder Cancer Evolution
Source: Adv Sci (Weinh). 2026 Oct 14:e78299. Online ahead of print. doi: 10.1002/advs.78299 (PMC13669223; doi:10.1002/advs.78299)
Supplement: Supplementary file 1 [file ADVS-9999-e78299-s001.docx]

| **Table S1. Operational definitions and construction of longitudinal paired cohorts.** | | | |
| --- | --- | --- | --- |
| Category | Item | Operational definition | Applied analysis |
| Longitudinal pair | Patient-level pair | Two temporally ordered pathological specimens from the same patient. | All longitudinal analyses |
| Time point | T0 specimen | The earlier available tumor specimen in a paired patient. | Baseline state |
| Time point | T1 specimen | The later available tumor specimen from the same patient. | Follow-up state |
| Pair-level change | Δ feature | Feature value at T1 minus feature value at T0. | Longitudinal drift analysis |
| Primary outcome | Progression | Documented pair-level pathological progression annotation reflecting pathological worsening from T0 to T1; the broad label was not reconstructed solely from derived stage/grade fields. | Primary progression label |
| Reference group | Non-progression | No documented progression between T0 and T1 during the paired interval. | Reference group |
| Sensitivity definition | Strict stage-or-grade progression | Derived progression requiring pathological stage or grade worsening from T0 to T1. | Sensitivity analysis |
| Sensitivity definition | Strict stage-only progression | Derived progression requiring pathological stage worsening from T0 to T1; grade-only upgrading was excluded. | Sensitivity analysis |
| Cohort construction | Eligible pair | Same patient identifier, valid T0/T1 WSI-derived features, and available progression status. | Final paired cohorts |
| Abbreviations: WSI, whole-slide image; Δ, T1 minus T0. Progression status was assigned at the pair level. Strict stage-or-grade and strict stage-only progression were applied only in sensitivity analyses. Eligible pairs required unambiguous patient linkage, valid T0/T1 WSI-derived features, and available progression status. | | | |

| **Table S2. Pair-level clinicopathological summary of the longitudinal cohorts.** | | | | | |
| --- | --- | --- | --- | --- | --- |
| Characteristic | | Center 1 non-progression (n = 51) | Center 1 progression (n = 35) | Center 2 non-progression (n = 58) | Center 2 progression (n = 38) |
| Number of paired cases | | 51 | 35 | 58 | 38 |
| Age at T0, years, median (IQR) | | 69 (59–77.5) | 77 (72.5–81) | 68.5 (63.0–72.0) | 69.5 (60.5–73.8) |
| Male sex, n (%) | | 42 (82.4) | 32 (91.4) | 53 (91.4) | 30 (78.9) |
| T0 pathological stage | Ta/Tis, n (%) | 30 (58.8) | 23 (65.7) | 22 (37.9) | 25 (65.8) |
|  | pT1, n (%) | 18 (35.3) | 10 (28.6) | 27 (46.6) | 8 (21.1) |
|  | ≥pT2, n (%) | 3 (5.9) | 2 (5.7) | 9 (15.5) | 5 (13.2) |
| T1 pathological stage | Ta/Tis, n (%) | 37 (72.5) | 9 (25.7) | 37 (63.8) | 6 (15.8) |
|  | pT1, n (%) | 14 (27.5) | 16 (45.7) | 18 (31.0) | 19 (50.0) |
|  | ≥pT2, n (%) | 0 (0.0) | 10 (28.6) | 3 (5.2) | 13 (34.2) |
| T0 high grade, n (%) | | 38 (74.5) | 23 (65.7) | 46 (79.3) | 22 (57.9) |
| T1 high grade, n (%) | | 32 (62.7) | 33 (94.3) | 39 (67.2) | 36 (94.7) |
| Stage upgrading, n (%) | | 0 (0.0) | 21 (60.0) | 0 (0.0) | 30 (78.9) |
| Grade upgrading, n (%) | | 0 (0.0) | 12 (34.3) | 0 (0.0) | 14 (36.8) |
| NMIBC-to-MIBC transition, n (%) | | 0 (0.0) | 10 (28.6) | 0 (0.0) | 10 (26.3) |
| Values are shown as median (IQR) or n (%). Progression status was assigned at the pair level. T0 denotes the earlier pathological specimen, and T1 denotes the later pathological specimen. T0 and T1 pathological stage rows refer to the stage category at each paired time point. Stage upgrading and grade upgrading were defined according to pathological change from T0 to T1. NMIBC-to-MIBC transition denotes progression from non-muscle-invasive to muscle-invasive bladder cancer between paired specimens. Abbreviations: IQR, interquartile range; MIBC, muscle-invasive bladder cancer; NMIBC, non-muscle-invasive bladder cancer. | | | | | |

| **Table S3. Slide-level and pair-level quality-control summary.** | | | | |
| --- | --- | --- | --- | --- |
| QC metric | Center 1 non-progression (n = 51) | Center 1 progression (n = 35) | Center 2 non-progression (n = 58) | Center 2 progression (n = 38) |
| Number of paired cases | 51 | 35 | 58 | 38 |
| Pairs retained in QC-enriched subset, n (%) | 48 (94.1) | 33 (94.3) | 58 (100.0) | 38 (100.0) |
| Pairs passing loose QC, n (%) | 48 (94.1) | 33 (94.3) | 58 (100.0) | 38 (100.0) |
| Any low nuclei-count flag, n (%) | 1 (2.0) | 1 (2.9) | 0 (0.0) | 0 (0.0) |
| Any low valid-patch flag, n (%) | 3 (5.9) | 1 (2.9) | 0 (0.0) | 0 (0.0) |
| Median nuclei count at T0, median (IQR) | 116895 (40093–387794.5) | 82465 (38526.5–224507) | 452876 (189682–820084) | 354506 (91072–511047) |
| Median nuclei count at T1, median (IQR) | 147719 (35367.5–272679) | 199870 (68132.5–362651.5) | 214166 (100403–352872) | 478197 (243276–657890) |
| Median valid patches at T0, median (IQR) | 214 (104.5–773.5) | 158 (94.5–522) | 737 (466–1160) | 596 (306–1003) |
| Median valid patches at T1, median (IQR) | 292 (91–637) | 490 (153.5–783.5) | 497 (251–907) | 786 (578–1044) |
| Median non-empty spatial bins at T0, median (IQR) | 214 (104.5–773.5) | 158 (94.5–522) | 737 (466–1160) | 596 (306–1003) |
| Median non-empty spatial bins at T1, median (IQR) | 292 (91–637) | 490 (153.5–783.5) | 497 (251–907) | 786 (578–1044) |
| Values are shown as n (%) or median (IQR). QC-enriched pairs were defined as pairs without low nuclei-count or low valid-patch flags at either time point. Loose QC pass was derived from the pair-level QC flag generated after T0/T1 matching. Valid patches and non-empty spatial bins refer to retained tissue-containing spatial units used for downstream aggregation. Abbreviations: QC, quality control; WSI, whole-slide image; T0, earlier pathological specimen; T1, later pathological specimen; IQR, interquartile range. | | | | |

| **Table S4. Complete feature definitions and aggregation rules.** | | | | |
| --- | --- | --- | --- | --- |
| Feature domain | Feature / metric | Definition | Aggregation level | Unit |
| Nuclear size | Nuclear area mean | Mean area of detected nuclei across the slide. | Slide level | μm² |
| Nuclear size | Nuclear area CV | Coefficient of variation of nuclear area across detected nuclei. | Slide level | Unitless |
| Nuclear size | Equivalent nuclear diameter mean | Mean area-derived equivalent circular diameter of detected nuclei, calculated for each segmented nuclear profile as $d_{eq}= \surd(4A/\pi)$, where A is nuclear area in μm². | Slide level | μm |
| Nuclear size | Equivalent nuclear diameter CV | Coefficient of variation of equivalent nuclear diameter across detected nuclei. | Slide level | Unitless |
| Shape regularity | Solidity mean | Mean ratio of nuclear area to convex-hull area. | Slide level | Unitless |
| Shape regularity | Solidity CV | Coefficient of variation of nuclear solidity across detected nuclei. | Slide level | Unitless |
| Shape regularity | Circularity mean | Mean nuclear circularity calculated as 4π × area / perimeter². | Slide level | Unitless |
| Shape regularity | Circularity CV | Coefficient of variation of nuclear circularity across detected nuclei. | Slide level | Unitless |
| Shape regularity | Aspect ratio mean | Mean major-axis length divided by minor-axis length. | Slide level | Unitless |
| Shape regularity | Aspect ratio CV | Coefficient of variation of nuclear aspect ratio across detected nuclei. | Slide level | Unitless |
| Local cellularity | Patch nuclei count mean | Mean number of retained nuclei across non-empty 500 × 500 μm spatial bins; not additionally normalized by tissue area. | Spatial-bin level | Nuclei per non-empty spatial bin |
| Local cellularity | Patch nuclei count CV | Coefficient of variation of retained-nuclei counts across non-empty 500 × 500 μm spatial bins. | Spatial-bin level | Unitless |
| Spatial organization | Spatial density mean | Mean across non-empty 500 × 500 μm spatial bins of D_j = n_j / 0.25 mm², where n_j is the retained-nucleus count in bin j. Normalization used the fixed full-bin area rather than measured tissue coverage. | Spatial-bin level | Nuclei/mm² |
| Spatial organization | Spatial density CV | Coefficient of variation of D_j = n_j / 0.25 mm² across non-empty 500 × 500 μm spatial bins. | Spatial-bin level | Unitless |
| Nuclear size | Patch-level nuclear-area CV | Coefficient of variation across non-empty 500 × 500 μm spatial bins of the bin-level mean nuclear area. | Spatial-bin level | Unitless |
| Shape regularity | Patch-level solidity CV | Coefficient of variation across non-empty 500 × 500 μm spatial bins of the bin-level mean nuclear solidity. | Spatial-bin level | Unitless |
| Shape regularity | Patch-level circularity CV | Coefficient of variation across non-empty 500 × 500 μm spatial bins of the bin-level mean nuclear circularity. | Spatial-bin level | Unitless |
| Shape regularity | Patch-level aspect-ratio CV | Coefficient of variation across non-empty 500 × 500 μm spatial bins of the bin-level mean nuclear aspect ratio. | Spatial-bin level | Unitless |
| Local cellularity | Mean nuclei-count shift | Mean nuclei count across non-empty 500-µm spatial bins at T1 minus the corresponding mean at T0. T0 and T1 spatial-bin distributions are summarized independently; no bin-level anatomical matching is assumed. | Patient-pair | Nuclei per non-empty spatial bin |
| Local cellularity | Positive nuclei-count quantile fraction | Fraction of prespecified quantiles (10th–90th percentiles) for which the T1 nuclei-count distribution exceeds the corresponding T0 quantile. | Patient-pair | Fraction |
| Nuclear size | Mean nuclear-area shift | Mean of spatial-bin mean nuclear area at T1 minus the corresponding mean at T0. T0 and T1 are summarized as separate within-slide distributions. | Patient-pair | µm² |
| Nuclear size | Positive nuclear-area quantile fraction | Fraction of prespecified quantiles (10th–90th percentiles) for which the T1 spatial-bin mean nuclear-area distribution exceeds the corresponding T0 quantile. | Patient-pair | Fraction |
| Spatial heterogeneity | Nuclear-area IQR shift | Interquartile range of spatial-bin mean nuclear area at T1 minus the corresponding interquartile range at T0. | Patient-pair | µm² |
| Summary axis | Nuclear size-related drift axis | Institutional IHC axis defined as the equal-weight mean of center-wise z-standardized Δ equivalent nuclear diameter mean and Δ nuclear area mean: A_NSD = [z(Δ equivalent nuclear diameter mean) + z(Δ nuclear area mean)] / 2. No PCA or fitted weighting was used. | Pair level | Center-standardized score |
| Summary axis | Exploratory drift score | Outcome-informed equal-weight mean of the top 10 Center 1 Δ features ranked by absolute Hedges' g, with Mann–Whitney U-test P value used only for secondary ordering. Selected features were z-standardized and direction-aligned using the sign of Hedges' g before averaging. No PCA or fitted weighting was used; the score was descriptive only. | Pair level | Standardized score |
| Summary axis | Nuclear size axis (TCGA) | Signed robust-z mean of area mean, area median, equivalent-diameter mean, equivalent-diameter median, and patch-area mean; all directions positive. Available components were equally weighted and the resulting axis was robust-standardized. | Slide level; patient-level mean | Robust-standardized score |
| Summary axis | Shape irregularity axis (TCGA) | Signed robust-z mean with negative directions for mean solidity and mean circularity, and positive directions for mean aspect ratio, solidity CV, circularity CV, aspect-ratio CV, patch-solidity-mean CV, patch-circularity-mean CV, and patch-aspect-mean CV. Available components were equally weighted and the resulting axis was robust-standardized. | Slide level; patient-level mean | Robust-standardized score |
| Summary axis | Spatial heterogeneity axis (TCGA) | Signed robust-z mean of patch nuclei-count CV, spatial-density CV, density CV, tile nuclei-count CV, tile-density CV, patch-area-mean CV, patch-solidity-mean CV, and patch-circularity-mean CV; all directions positive. Available components were equally weighted and the resulting axis was robust-standardized. | Slide level; patient-level mean | Robust-standardized score |
| Summary axis | Composite morphology axis (TCGA) | Equal-weight mean of the TCGA nuclear size, shape irregularity, and spatial heterogeneity axes, followed by robust standardization. | Slide level; patient-level mean | Robust-standardized score |
| Abbreviations: CV, coefficient of variation; WSI, whole-slide image; Δ, T1 minus T0; MAD, median absolute deviation; IHC, immunohistochemistry. Pair-level Δ features were calculated as T1 minus T0. Tumor-restricted analyses retained only nuclei within pathologist-confirmed tumor ROIs, and T0/T1 spatial-bin distributions were summarized independently without anatomical matching. Patch nuclei-count features represent local nuclear abundance rather than tissue-area-normalized density. Spatial density was expressed as retained nuclei per fixed 500 × 500 μm bin area (0.25 mm²), not per measured tissue-covered area. For summary axes, component features were standardized within the relevant dataset, direction-aligned where specified, equally weighted, and averaged; no PCA or fitted coefficients were used. TCGA axes used median/MAD-based robust z-standardization, and multiple WSIs from the same patient were averaged at the patient level. | | | | |

| **Table S5. Full feature-level longitudinal drift results across Centers 1 and 2.** | | | | | |
| --- | --- | --- | --- | --- | --- |
| Center | Feature domain | Pair-level Δ feature | Non-progression median (IQR) | Progression median (IQR) | Effect summary |
| Center 1 | Local cellularity / spatial organization | Δ spatial density CV | 0.04 (-0.15 to 0.2) | -0.07 (-0.26 to 0.06) | SMD = -0.522 (95% CI -0.96 to -0.08); P = 0.023; FDR = 0.203 |
| Center 1 | Local cellularity / spatial organization | Δ patch nuclei-count CV | 0.04 (-0.15 to 0.2) | -0.07 (-0.26 to 0.06) | SMD = -0.522 (95% CI -0.96 to -0.08); P = 0.023; FDR = 0.203 |
| Center 1 | Nuclear size | Δ equivalent nuclear diameter CV | 0.01 (-0.02 to 0.05) | 0.02 (-0.03 to 0.05) | SMD = 0.212 (95% CI -0.22 to 0.64); P = 0.972; FDR = 0.972 |
| Center 1 | Nuclear size | Δ nuclear area CV | 0.02 (-0.14 to 0.12) | 0.03 (-0.03 to 0.13) | SMD = 0.232 (95% CI -0.20 to 0.66); P = 0.389; FDR = 0.849 |
| Center 1 | Shape regularity | Δ solidity CV | 0 (-0.01 to 0) | 0 (-0.01 to 0.01) | SMD = 0.186 (95% CI -0.25 to 0.62); P = 0.424; FDR = 0.849 |
| Center 1 | Local cellularity / spatial organization | Δ spatial density mean | -19.71 (-1013.53 to 556.85) | 0 (-733.38 to 602.59) | SMD = 0.168 (95% CI -0.26 to 0.60); P = 0.755; FDR = 0.849 |
| Center 1 | Local cellularity / spatial organization | Δ patch nuclei count mean | -4.93 (-253.38 to 139.21) | 0 (-183.34 to 150.65) | SMD = 0.168 (95% CI -0.26 to 0.60); P = 0.755; FDR = 0.849 |
| Center 1 | Shape regularity | Δ circularity mean | -0.03 (-0.1 to 0.05) | -0.03 (-0.08 to 0.01) | SMD = -0.149 (95% CI -0.58 to 0.28); P = 0.616; FDR = 0.849 |
| Center 1 | Nuclear size | Δ nuclear area mean | 0.73 (-5.03 to 9.8) | 0.57 (-7.35 to 10.3) | SMD = 0.097 (95% CI -0.33 to 0.53); P = 0.843; FDR = 0.893 |
| Center 1 | Shape regularity | Δ aspect ratio mean | 0 (-0.13 to 0.14) | 0.09 (-0.18 to 0.25) | SMD = -0.112 (95% CI -0.54 to 0.32); P = 0.321; FDR = 0.849 |
| Center 1 | Shape regularity | Δ solidity mean | 0.01 (-0.01 to 0.01) | 0 (-0.01 to 0.01) | SMD = -0.125 (95% CI -0.56 to 0.31); P = 0.504; FDR = 0.849 |
| Center 1 | Shape regularity | Δ circularity CV | -0 (-0.02 to 0.02) | 0 (-0.02 to 0.04) | SMD = -0.057 (95% CI -0.49 to 0.37); P = 0.270; FDR = 0.849 |
| Center 1 | Shape regularity | Δ patch-level aspect-ratio CV | 0 (-0.12 to 0.05) | -0 (-0.02 to 0.06) | SMD = 0.082 (95% CI -0.35 to 0.51); P = 0.530; FDR = 0.849 |
| Center 1 | Shape regularity | Δ aspect ratio CV | 0.01 (-0.06 to 0.06) | 0.05 (-0.03 to 0.11) | SMD = 0.075 (95% CI -0.36 to 0.50); P = 0.078; FDR = 0.468 |
| Center 1 | Shape regularity | Δ patch-level circularity CV | -0 (-0.04 to 0.02) | -0 (-0.02 to 0.02) | SMD = -0.050 (95% CI -0.48 to 0.38); P = 0.553; FDR = 0.849 |
| Center 1 | Shape regularity | Δ patch-level solidity CV | -0 (-0.01 to 0) | -0 (-0 to 0) | SMD = 0.059 (95% CI -0.37 to 0.49); P = 0.735; FDR = 0.849 |
| Center 1 | Nuclear size | Δ equivalent nuclear diameter mean | 0.11 (-0.61 to 1.32) | 0.1 (-0.9 to 1.3) | SMD = -0.048 (95% CI -0.48 to 0.38); P = 0.686; FDR = 0.849 |
| Center 1 | Nuclear size | Δ patch-level nuclear-area CV | -0.03 (-1.33 to 0.11) | -0 (-0.39 to 0.16) | SMD = -0.051 (95% CI -0.48 to 0.38); P = 0.748; FDR = 0.849 |
| Center 2 | Nuclear size | Δ equivalent nuclear diameter mean | -0.514 (-0.823 to 0.070) | 0.324 (-0.133 to 0.617) | SMD = 0.716 (95% CI 0.29 to 1.14); P < 0.001; FDR = 0.004 |
| Center 2 | Nuclear size | Δ nuclear area mean | -4.29 (-7.90 to 0.743) | 2.73 (-1.88 to 6.12) | SMD = 0.685 (95% CI 0.26 to 1.11); P < 0.001; FDR = 0.004 |
| Center 2 | Shape regularity | Δ solidity mean | -0.002 (-0.008 to 0.003) | 0.001 (-0.001 to 0.006) | SMD = 0.488 (95% CI 0.07 to 0.90); P = 0.012; FDR = 0.075 |
| Center 2 | Shape regularity | Δ solidity CV | 0.003 (-0.002 to 0.010) | -0.000 (-0.006 to 0.003) | SMD = -0.417 (95% CI -0.831 to -0.004); P = 0.036; FDR = 0.115 |
| Center 2 | Shape regularity | Δ patch-level solidity CV | -0.001 (-0.012 to 0.006) | 0.001 (-0.005 to 0.014) | SMD = 0.351 (95% CI -0.06 to 0.76); P = 0.115; FDR = 0.207 |
| Center 2 | Local cellularity / spatial organization | Δ patch nuclei count mean | -79.7 (-282.5 to 155.5) | 121.5 (-139.9 to 237.6) | SMD = 0.337 (95% CI -0.08 to 0.75); P = 0.057; FDR = 0.127 |
| Center 2 | Local cellularity / spatial organization | Δ spatial density mean | -318.7 (-1130.0 to 621.9) | 485.8 (-559.5 to 950.5) | SMD = 0.337 (95% CI -0.08 to 0.75); P = 0.057; FDR = 0.127 |
| Center 2 | Local cellularity / spatial organization | Δ patch nuclei-count CV | 0.053 (-0.227 to 0.377) | -0.134 (-0.286 to 0.105) | SMD = -0.305 (95% CI -0.72 to 0.11); P = 0.038; FDR = 0.115 |
| Center 2 | Local cellularity / spatial organization | Δ spatial density CV | 0.053 (-0.227 to 0.377) | -0.134 (-0.286 to 0.105) | SMD = -0.305 (95% CI -0.72 to 0.11); P = 0.038; FDR = 0.115 |
| Center 2 | Nuclear size | Δ equivalent nuclear diameter CV | 0.001 (-0.022 to 0.026) | 0.006 (-0.008 to 0.028) | SMD = 0.240 (95% CI -0.17 to 0.65); P = 0.412; FDR = 0.570 |
| Center 2 | Shape regularity | Δ circularity CV | 0.007 (-0.022 to 0.037) | -0.004 (-0.023 to 0.027) | SMD = -0.221 (95% CI -0.63 to 0.19); P = 0.355; FDR = 0.532 |
| Center 2 | Shape regularity | Δ aspect ratio mean | 0.021 (-0.128 to 0.157) | -0.017 (-0.166 to 0.124) | SMD = 0.182 (95% CI -0.23 to 0.59); P = 0.551; FDR = 0.709 |
| Center 2 | Shape regularity | Δ circularity mean | 0.008 (-0.019 to 0.030) | 0.001 (-0.023 to 0.022) | SMD = -0.158 (95% CI -0.57 to 0.25); P = 0.328; FDR = 0.532 |
| Center 2 | Nuclear size | Δ nuclear area CV | 0.018 (-0.089 to 0.109) | -0.003 (-0.056 to 0.077) | SMD = 0.113 (95% CI -0.30 to 0.52); P = 0.634; FDR = 0.750 |
| Center 2 | Shape regularity | Δ patch-level circularity CV | -0.001 (-0.024 to 0.023) | 0.002 (-0.027 to 0.018) | SMD = 0.063 (95% CI -0.35 to 0.47); *P* = 0.985; FDR = 0.985 |
| Center 2 | Shape regularity | Δ aspect ratio CV | 0.004 (-0.060 to 0.065) | 0.023 (-0.043 to 0.086) | SMD = 0.022 (95% CI -0.39 to 0.43); *P* = 0.667; FDR = 0.750 |
| Center 2 | Shape regularity | Δ patch-level aspect-ratio CV | -0.065 (-0.538 to 0.085) | 0.024 (-0.173 to 0.183) | SMD = -0.018 (95% CI -0.43 to 0.39); P = 0.091; FDR = 0.182 |
| Center 2 | Nuclear size | Δ patch-level nuclear-area CV | -0.147 (-1.35 to 0.814) | -0.009 (-0.847 to 0.639) | SMD = 0.014 (95% CI -0.40 to 0.42); *P* = 0.802; FDR = 0.849 |
| Values are median (IQR). Pair-level Δ features were calculated as T1 minus T0. SMD represents standardized mean difference comparing progression with non-progression pairs; positive values indicate higher Δ values in progression pairs. P values were calculated using two-sided Mann–Whitney U tests and FDR by Benjamini–Hochberg correction. CV, coefficient of variation; FDR, false discovery rate; IQR, interquartile range; SMD, standardized mean difference. All Center 1 comparisons used 35 progression and 51 non-progression pairs; all Center 2 comparisons used 38 progression and 58 non-progression pairs. SMDs are reported as Hedges' g with 95% confidence intervals. | | | | | |

| **Table S6. Sensitivity and adjusted association analyses.** | | | | | |
| --- | --- | --- | --- | --- | --- |
| Analysis category | Center | Feature / metric | Analysis set / model | Progression / non-progression (n) | Effect summary |
| QC sensitivity | Center 1 | Δ spatial density CV | All pairs | 35/51 | SMD -0.52 (95% CI -0.96 to -0.08); P = 0.023 |
| QC sensitivity | Center 1 | Δ spatial density CV | QC-enriched subset | 33/48 | SMD -0.42 (95% CI -0.87 to 0.03); P = 0.096 |
| QC sensitivity | Center 2 | Δ equivalent nuclear diameter mean | All pairs | 38/58 | SMD 0.72 (95% CI 0.29 to 1.14); P < 0.001 |
| QC sensitivity | Center 2 | Δ equivalent nuclear diameter mean | QC-enriched subset | 38/58 | SMD 0.72 (95% CI 0.29 to 1.14); P < 0.001 |
| QC sensitivity | Center 2 | Δ nuclear area mean | All pairs | 38/58 | SMD 0.68 (95% CI 0.26 to 1.11); P < 0.001 |
| QC sensitivity | Center 2 | Δ nuclear area mean | QC-enriched subset | 38/58 | SMD 0.68 (95% CI 0.26 to 1.11); P < 0.001 |
| Progression-definition sensitivity | Center 1 | Δ spatial density CV | Broad progression | 35/51 | SMD -0.52 (95% CI -0.96 to -0.08); P = 0.023 |
| Progression-definition sensitivity | Center 1 | Δ spatial density CV | Strict stage-only progression | 21/51 | SMD -0.64 (95% CI -1.16 to -0.13); P = 0.023 |
| Progression-definition sensitivity | Center 2 | Δ equivalent nuclear diameter mean | Broad (combined) progression | 38/58 | SMD (Hedges' g) = 0.72 (95% CI 0.29–1.14); *P* < 0.001 |
| Progression-definition sensitivity | Center 2 | Δ equivalent nuclear diameter mean | Strict stage-only progression | 30/58 | SMD (Hedges' g) = 0.63 (95% CI 0.18–1.09); *P* = 0.003 |
| Progression-definition sensitivity | Center 2 | Δ nuclear area mean | Broad (combined) progression | 38/58 | SMD (Hedges' g) = 0.68 (95% CI 0.26–1.11); *P* < 0.001 |
| Progression-definition sensitivity | Center 2 | Δ nuclear area mean | Strict stage-only progression | 30/58 | SMD (Hedges' g) = 0.60 (95% CI 0.15–1.05); *P* = 0.003 |
| Progression-definition sensitivity | Center 2 | Δ equivalent nuclear diameter mean | NMIBC-to-MIBC transition | 10/58 | SMD (Hedges' g) = 0.86 (95% CI 0.17–1.55); *P* = 0.021 |
| Progression-definition sensitivity | Center 2 | Δ nuclear area mean | NMIBC-to-MIBC transition | 10/58 | SMD (Hedges' g) = 0.86 (95% CI 0.18–1.55); *P* = 0.025 |
| Progression-definition sensitivity | Center 2 | Δ equivalent nuclear diameter mean | Grade-only upgrading | 8/58 | SMD (Hedges' g) = 1.06 (95% CI 0.30–1.82); *P* = 0.004 |
| Progression-definition sensitivity | Center 2 | Δ nuclear area mean | Grade-only upgrading | 8/58 | SMD (Hedges' g) = 1.06 (95% CI 0.30–1.82); *P* = 0.006 |
| Adjusted association | Center 1 | Δ spatial density CV | Age + sex + MIBC status | 35/51 | OR 0.58 (0.35–0.96); P = 0.033 |
| Adjusted association | Center 2 | Δ equivalent nuclear diameter mean | Age + sex + MIBC status | 38/58 | OR 2.43 (1.46–4.06); *P* < 0.001 |
| Adjusted association | Center 2 | Δ nuclear area mean | Age + sex + MIBC status | 38/58 | OR 2.38 (1.42–3.99); *P* < 0.001 |
| Adjusted association | Center 2 | Δ solidity mean | Age + sex + MIBC status | 38/58 | OR 1.76 (1.10–2.82); *P* = 0.018 |
| Tumor-region sensitivity | Center 1 | Δ nuclear area mean | Whole-tissue | 35/51 | SMD (Hedges' g) = 0.10 (95% CI -0.33–0.53); P = 0.843 |
| Tumor-region sensitivity | Center 1 | Δ nuclear area mean | Pathologist-confirmed tumor-only | 35/51 | SMD (Hedges' g) = 0.25 (95% CI -0.18–0.69); P = 0.414 |
| Tumor-region sensitivity | Center 1 | Δ equivalent nuclear diameter mean | Whole-tissue | 35/51 | SMD (Hedges' g) = -0.05 (95% CI -0.48–0.38); P = 0.686 |
| Tumor-region sensitivity | Center 1 | Δ equivalent nuclear diameter mean | Pathologist-confirmed tumor-only | 35/51 | SMD (Hedges' g) = 0.09 (95% CI -0.34–0.52); P = 0.568 |
| Tumor-region sensitivity | Center 2 | Δ nuclear area mean | Whole-tissue | 38/58 | SMD (Hedges' g) = 0.68 (95% CI 0.26–1.11); P < 0.001 |
| Tumor-region sensitivity | Center 2 | Δ nuclear area mean | Pathologist-confirmed tumor-only | 38/58 | SMD (Hedges' g) = 0.52 (95% CI 0.10–0.93); P = 0.013 |
| Tumor-region sensitivity | Center 2 | Δ equivalent nuclear diameter mean | Whole-tissue | 38/58 | SMD (Hedges' g) = 0.72 (95% CI 0.29–1.14); P < 0.001 |
| Tumor-region sensitivity | Center 2 | Δ equivalent nuclear diameter mean | Pathologist-confirmed tumor-only | 38/58 | SMD (Hedges' g) = 0.50 (95% CI 0.08–0.91); P = 0.011 |
| Abbreviations: CI, confidence interval; CV, coefficient of variation; MIBC, muscle-invasive bladder cancer; OR, odds ratio; SMD, standardized mean difference; Δ, T1 minus T0. The table presents selected key sensitivity and adjusted analyses; full forest plots are shown in Supplementary Figure S3. Tumor-region sensitivity analyses compare whole-tissue and pathologist-confirmed tumor-only feature extraction. Effect sizes for this analysis are standardized mean differences reported as Hedges' g with 95% confidence intervals. For QC and progression-definition sensitivity analyses, SMDs are also reported as Hedges' g with 95% confidence intervals. Feature-level FDR values are reported in Table S5 and are not repeated in this selected sensitivity-summary table. | | | | | |

| **Table S7. Cross-backbone performance of static WSI models under internal and locked external validation.** | | | | | |
| --- | --- | --- | --- | --- | --- |
| **Validation setting** | **Validation cohort** | **Encoder** | **Clinical-only AUC** | **Pathology-only AUC** | **Fusion AUC** |
| Internal validation | Center 1 | UNI2 | 0.72 (0.67–0.77) | 0.69 (0.65–0.74) | 0.75 (0.71–0.79) |
| Internal validation | Center 1 | CTransPath | 0.72 (0.67–0.77) | 0.62 (0.57–0.67) | 0.72 (0.68–0.77) |
| Internal validation | Center 1 | ResNet50 | 0.72 (0.67–0.77) | 0.69 (0.64–0.73) | 0.75 (0.70–0.79) |
| Internal validation | Center 1 | DenseNet121 | 0.72 (0.67–0.77) | 0.67 (0.63–0.72) | 0.74 (0.69–0.78) |
| Locked external validation | Center 2 | UNI2 | 0.53 (0.47–0.58) | 0.70 (0.66–0.75) | 0.65 (0.60–0.69) |
| Locked external validation | Center 2 | CTransPath | 0.53 (0.47–0.58) | 0.52 (0.47–0.56) | 0.53 (0.48–0.58) |
| Locked external validation | Center 2 | ResNet50 | 0.53 (0.47–0.58) | 0.67 (0.62–0.71) | 0.60 (0.55–0.65) |
| Locked external validation | Center 2 | DenseNet121 | 0.53 (0.47–0.58) | 0.67 (0.63–0.72) | 0.60 (0.56–0.65) |
| Locked external validation | Center 3 | UNI2 | 0.72 (0.61–0.82) | 0.71 (0.61–0.80) | 0.78 (0.69–0.87) |
| Locked external validation | Center 3 | CTransPath | 0.72 (0.61–0.82) | 0.62 (0.51–0.73) | 0.71 (0.61–0.80) |
| Locked external validation | Center 3 | ResNet50 | 0.72 (0.61–0.82) | 0.67 (0.58–0.77) | 0.76 (0.66–0.85) |
| Locked external validation | Center 3 | DenseNet121 | 0.72 (0.61–0.82) | 0.73 (0.64–0.82) | 0.79 (0.70–0.88) |
| Locked external validation | Center 4 | UNI2 | 0.58 (0.49–0.67) | 0.64 (0.54–0.73) | 0.61 (0.53–0.71) |
| Locked external validation | Center 4 | CTransPath | 0.58 (0.49–0.67) | 0.59 (0.49–0.69) | 0.61 (0.52–0.70) |
| Locked external validation | Center 4 | ResNet50 | 0.58 (0.49–0.67) | 0.63 (0.53–0.71) | 0.62 (0.53–0.70) |
| Locked external validation | Center 4 | DenseNet121 | 0.58 (0.49–0.67) | 0.64 (0.54–0.73) | 0.63 (0.54–0.71) |
| Values are AUCs with 95% confidence intervals. Center 1 internal performance was estimated using repeated stratified cross-validation. For Centers 2–4, all preprocessing transformations and fitted models were derived from Center 1 and applied without center-specific retraining or recalibration. Clinical-only performance is repeated within each encoder block to facilitate direct comparison. AUC, area under the receiver operating characteristic curve; CI, confidence interval; WSI, whole-slide image. | | | | | |

| **Table S8. IHC and TCGA biological anchoring results.** | | | | | |
| --- | --- | --- | --- | --- | --- |
| Anchoring layer | Analysis | Feature / axis | Biological readout | Result summary | Interpretation |
| IHC anchoring | IHC subset availability | Combined Center 1/2 IHC subset | Ki-67 and HER2 measurements | Any IHC n = 91; Ki-67 endpoint n = 85; ΔKi-67 n = 44; HER2 endpoint n = 63. | Defines the available tissue-level anchoring cohort. |
| IHC anchoring | Primary Ki-67 endpoint correlation | Nuclear size-related drift axis | T1 Ki-67 index | Center 2: Spearman ρ = 0.267, *P* = 0.0468, n = 56. | Modest positive tissue-level proliferation anchoring. |
| IHC anchoring | Longitudinal Ki-67 change by progression | Progression status | ΔKi-67 index | Center 2: progression median = 20.0%, non-progression median = -10.0%, *P* = 0.0348; n = 12/16. | Progression pairs showed a greater increase in Ki-67. |
| IHC anchoring | Ki-67 correlation with nuclear-size feature | Δ equivalent nuclear diameter mean | T1 Ki-67 index | Center 2: Spearman ρ = 0.263, *P* = 0.0502, n = 56. | Concordant supportive trend. |
| IHC anchoring | Ki-67 correlation with nuclear-size feature | Δ nuclear area mean | T1 Ki-67 index | Center 2: Spearman ρ = 0.254, *P* = 0.0590, n = 56. | Concordant supportive trend. |
| IHC anchoring | Exploratory HER2 analysis | Nuclear size-related drift axis | Ordinal HER2 IHC score | Center 2: Kruskal–Wallis *P* = 0.1363, n = 41; 4 HER2 score categories. | Exploratory; no consistent ordinal group pattern. |
| TCGA anchoring | TCGA sample availability | Pathology-derived axes | TCGA-BLCA transcriptomic data | Common patient-level pathology and transcriptomic data: n = 380. | Used for molecular anchoring, not longitudinal validation. |
| TCGA anchoring | Immune signature correlation | Nuclear size axis | Immune signature scores | CD8 T cells ρ = -0.268, FDR = 4.62e-06; cytolytic activity ρ = -0.260, FDR = 5.55e-06; T-cell inflamed ρ = -0.237, FDR = 2.89e-05. | Inverse association with immune-inflamed signatures. |
| TCGA anchoring | Hallmark pathway correlation | Nuclear size axis | Hallmark pathway scores | Allograft rejection ρ = -0.206, FDR = 0.0109; IFN-γ response ρ = -0.184, FDR = 0.0310. | Inverse association with inflammatory pathway activity. |
| TCGA anchoring | Gene-level association | Nuclear size axis | Axis-associated genes | Top immune genes included ZAP70 (ρ = -0.334, FDR = 6.13e-07), TBC1D10C (ρ = -0.332, FDR = 6.13e-07), and CD3D (ρ = -0.317, FDR = 2.65e-06). | Gene-level associations were concordant with immune-related anchoring. |
| TCGA anchoring | Hallmark ORA | Nuclear size axis | Negative axis-associated genes | Allograft rejection OR = 36.1, FDR = 1.10e-76; IFN-γ response OR = 19.8, FDR = 1.85e-41. | Negative axis-associated genes were enriched for immune programs. |
| TCGA anchoring | Hallmark ORA | Shape irregularity axis | Positive axis-associated genes | MTORC1 signaling OR = 6.3, FDR = 0.0119; xenobiotic metabolism OR = 6.3, FDR = 0.0119. | Exploratory pathway enrichment for shape-related axis. |
| TCGA anchoring | Hallmark ORA | Spatial heterogeneity axis | Positive axis-associated genes | E2F targets OR = 42.5, FDR = 1.76e-57; G2M checkpoint OR = 29.5, FDR = 3.14e-39. | Strong enrichment for cell-cycle programs. |
| TCGA anchoring | Hallmark ORA | Composite morphology axis | Negative axis-associated genes | Allograft rejection OR = 42.9, FDR = 3.85e-27; IFN-γ response OR = 16.3, FDR = 1.51e-08. | Composite axis showed immune/inflammatory pathway anchoring. |
| Abbreviations: BLCA, bladder urothelial carcinoma; FDR, false discovery rate; HER2, human epidermal growth factor receptor 2; IHC, immunohistochemistry; IFN-γ, interferon gamma; OR, odds ratio; ORA, over-representation analysis; TCGA, The Cancer Genome Atlas; Δ, T1 minus T0. IHC analyses were exploratory tissue-level anchoring analyses. TCGA analyses were used for molecular anchoring of pathology-derived axes and not as direct longitudinal validation. | | | | | |

| **Table S9. Technical validation and sensitivity analyses for nuclear segmentation** | | | |
| --- | --- | --- | --- |
| **Analysis** | **Cohort** | **Evaluation set / parameter** | **Result** |
| **MPP and scale audit** | Center 1 | Longitudinal WSIs, n | 172 |
|  | Center 1 | Native/recovered-scale WSIs, n (%) | 139 (80.8%) |
|  | Center 1 | Final fallback-scaled WSIs, n (%) | 33 (19.2%) |
|  | Center 1 | Unresolved scale, n | 0 |
|  | Center 2 | Longitudinal WSIs, n | 192 |
|  | Center 2 | Native/recovered-scale WSIs, n (%) | 192 (100.0%) |
|  | Center 2 | Final fallback-scaled WSIs, n (%) | 0 (0.0%) |
|  | Center 2 | Unresolved scale, n | 0 |
| **Manual segmentation validation** | Center 1 | Validation windows, n | 6 |
|  | Center 1 | Reviewed nuclei, n | 1,056 |
|  | Center 1 | Pixel-level precision | 0.620 |
|  | Center 1 | Pixel-level recall | 0.546 |
|  | Center 1 | Dice coefficient | 0.581 |
|  | Center 1 | IoU | 0.409 |
|  | Center 2 | Validation windows, n | 6 |
|  | Center 2 | Reviewed nuclei, n | 1,437 |
|  | Center 2 | Pixel-level precision | 0.846 |
|  | Center 2 | Pixel-level recall | 0.606 |
|  | Center 2 | Dice coefficient | 0.706 |
|  | Center 2 | IoU | 0.546 |
| **Cellpose parameter sensitivity** | Center 2 | Longitudinal cohort | 96 pairs / 192 WSIs |
|  | Center 2 | Nominal nuclear diameter | 12, 15, and 18 pixels |
|  | Center 2 | Minimum nuclear-area threshold | 5 and 10 μm² |
|  | Center 2 | Parameter combinations examined | 6 |
|  | Center 2 | Nuclear-size readouts examined | 4 |
|  | Center 2 | Direction preserved vs. D15/5-μm² baseline | 20/20 comparisons |
| **Border-removal sensitivity** | Center 1 | Validation windows | No-border/border count ratio = 1.00 |
|  | Center 2 | Validation windows | No-border/border count ratio = 1.00 |
| Abbreviations: MPP, microns per pixel; WSI, whole-slide image; IoU, intersection over union. MPP and coordinate scale were audited for all 364 longitudinal WSIs; no WSI remained unresolved after audit. Segmentation accuracy was evaluated against manually reviewed nuclear masks in 12 stratified tumor-region windows, with six windows from each center. Pixel-level precision, recall, Dice coefficient, and IoU were calculated from foreground overlap. Parameter sensitivity was assessed in the complete Center 2 longitudinal cohort using Cellpose diameters of 12, 15, and 18 pixels and minimum nuclear-area thresholds of 5 and 10 μm². The four nuclear-size readouts were mean and median nuclear area and mean and median area-derived equivalent nuclear diameter. Border-removal sensitivity was evaluated in the same manually reviewed regions.  After the final Center 1 VSI reconstruction, 35 WSIs were reprocessed from the original VSI files with native MPP. The final post-rebuild audit therefore classified 139/172 Center 1 WSIs as native/recovered-scale and 33/172 as fallback-scaled. None of the 192 Center 2 WSIs required fallback scaling. These post-rebuild counts replace the earlier pre-reconstruction 59-slide coded-scale count. | | | |

| **Table S10. Distribution of fallback-scaled slides and sensitivity analysis after excluding affected pairs** | | | |
| --- | --- | --- | --- |
| **Analysis** | **Cohort** | **Evaluation set / measure** | **Result** |
| **Final fallback-scale distribution** | Center 1 | All longitudinal WSIs | 33/172 (19.2%) |
|  | Center 1 | T0 WSIs | 20/86 (23.3%) |
|  | Center 1 | T1 WSIs | 13/86 (15.1%) |
|  | Center 2 | All longitudinal WSIs | 0/192 (0.0%) |
|  | Center 2 | T0 WSIs | 0/96 (0.0%) |
|  | Center 2 | T1 WSIs | 0/96 (0.0%) |
| **Pairs containing ≥1 fallback-scaled WSI** | Center 1 | All pairs | 30/86 (34.9%) |
|  | Center 1 | Progression | 13/35 (37.1%) |
|  | Center 1 | Non-progression | 17/51 (33.3%) |
|  | Center 1 | Fallback pattern | T0 only 17; T1 only 10; both 3 |
|  | Center 1 | Progression-group balance | Fisher's exact P = 0.819 |
|  | Center 1 | T0-T1 imbalance | Exact paired P = 0.248 |
|  | Center 2 | All pairs | 0/96 (0.0%) |
| **Fallback-exclusion sensitivity - whole tissue** | Center 1 | Δ nuclear area mean | g = 0.08 (95% CI -0.46 to 0.61); P = 0.960; FDR = 0.960 |
|  | Center 1 | Δ nuclear area median | g = 0.05 (95% CI -0.48 to 0.59); P = 0.933; FDR = 0.960 |
|  | Center 1 | Δ equivalent nuclear diameter mean | g = 0.04 (95% CI -0.49 to 0.58); P = 0.927; FDR = 0.960 |
|  | Center 1 | Δ equivalent nuclear diameter median | g = 0.03 (95% CI -0.51 to 0.57); P = 0.920; FDR = 0.960 |
|  | Center 2 | Δ nuclear area mean | g = 0.68 (95% CI 0.26 to 1.11); P = 0.0004; FDR = 0.0004 |
|  | Center 2 | Δ nuclear area median | g = 0.65 (95% CI 0.23 to 1.07); P = 0.0002; FDR = 0.0004 |
|  | Center 2 | Δ equivalent nuclear diameter mean | g = 0.72 (95% CI 0.29 to 1.14); P = 0.0004; FDR = 0.0004 |
|  | Center 2 | Δ equivalent nuclear diameter median | g = 0.69 (95% CI 0.27 to 1.11); P = 0.0003; FDR = 0.0004 |
| **Fallback-exclusion sensitivity - tumor-only** | Center 1 | Δ nuclear area mean | g = 0.43 (95% CI -0.11 to 0.97); P = 0.244; FDR = 0.476 |
|  | Center 1 | Δ equivalent nuclear diameter mean | g = 0.36 (95% CI -0.18 to 0.90); P = 0.326; FDR = 0.476 |
|  | Center 2 | Δ nuclear area mean | g = 0.52 (95% CI 0.10 to 0.93); P = 0.013; FDR = 0.019 |
|  | Center 2 | Δ equivalent nuclear diameter mean | g = 0.50 (95% CI 0.08 to 0.91); P = 0.011; FDR = 0.019 |
| Abbreviations: WSI, whole-slide image; g, Hedges' g; CI, confidence interval; FDR, false discovery rate. After the final slide-level MPP audit and Center 1 VSI reconstruction, we classified a slide as fallback-scaled only when metadata review could not recover a reliable native scale and the predefined 0.25 μm/pixel value remained necessary. All 33 final fallback-scaled WSIs came from Center 1. For the exclusion analysis, we removed the entire T0-T1 pair whenever either slide required fallback scaling, leaving 56 Center 1 pairs (22 progression and 34 non-progression). Center 2 contained no fallback-scaled slides, so its estimates did not change. P values are from two-sided Mann-Whitney U tests. We calculated FDR values across the four prespecified nuclear-size readouts within each cohort and analysis set. | | | |

| **Table S11. Treatment exposure, T0–T1 interval, procedure type, and restricted sensitivity analyses.** | | | |
| --- | --- | --- | --- |
| **Analysis** | **Cohort** | **Evaluation set / measure** | **Result** |
| **T0–T1 interval distribution** | Center 1 | All pairs | Median 16 months (IQR 7.25–28); range 2–69 |
|  | Center 1 | Non-progression | Median 20 months (IQR 9.5–34) |
|  | Center 1 | Progression | Median 12 months (IQR 6.5–21.5) |
|  | Center 1 | Progression vs non-progression | Two-sided Mann–Whitney U test: P = 0.057 |
|  | Center 2 | All pairs | Median 12.5 months (IQR 7–27); range 1–95 |
|  | Center 2 | Non-progression | Median 12 months (IQR 6–17.75) |
|  | Center 2 | Progression | Median 13 months (IQR 8.5–34) |
|  | Center 2 | Progression vs non-progression | Two-sided Mann–Whitney U test: P = 0.104 |
| **Intercurrent postoperative treatment after T0** | Center 1 | Non-progression | Intravesical chemotherapy: 42/51 (82.4%); Systemic chemotherapy / immunotherapy: 8/51 (15.7%); None: 1/51 (2.0%) |
|  | Center 1 | Progression | Intravesical chemotherapy: 26/35 (74.3%); Systemic chemotherapy / immunotherapy: 5/35 (14.3%); None: 4/35 (11.4%) |
|  | Center 2 | Non-progression | Intravesical chemotherapy: 50/58 (86.2%); BCG: 1/58 (1.7%); Systemic chemotherapy / immunotherapy: 4/58 (6.9%); None: 3/58 (5.2%) |
|  | Center 2 | Progression | Intravesical chemotherapy: 30/38 (78.9%); BCG: 2/38 (5.3%); Systemic chemotherapy / immunotherapy: 2/38 (5.3%); None: 3/38 (7.9%); Other / unknown: 1/38 (2.6%) |
|  | Center 1 | Intravesical-treatment balance | Progression 26/35 vs non-progression 42/51; Fisher's exact P = 0.424 |
|  | Center 2 | Intravesical-treatment balance | Progression 30/38 vs non-progression 50/58; Fisher's exact P = 0.407 |
| **Procedure type at T0/T1** | Center 1 | T0, Non-progression | TURBT: 45/51 (88.2%); Radical cystectomy: 1/51 (2.0%); Partial cystectomy: 5/51 (9.8%) |
|  | Center 1 | T0, Progression | TURBT: 33/35 (94.3%); Radical cystectomy: 1/35 (2.9%); Partial cystectomy: 1/35 (2.9%) |
|  | Center 1 | T1, Non-progression | TURBT: 49/51 (96.1%); Radical cystectomy: 2/51 (3.9%) |
|  | Center 1 | T1, Progression | TURBT: 25/35 (71.4%); Radical cystectomy: 7/35 (20.0%); Partial cystectomy: 3/35 (8.6%) |
|  | Center 2 | T0, Non-progression | TURBT: 53/58 (91.4%); Partial cystectomy: 5/58 (8.6%) |
|  | Center 2 | T0, Progression | TURBT: 37/38 (97.4%); Partial cystectomy: 1/38 (2.6%) |
|  | Center 2 | T1, Non-progression | TURBT: 52/58 (89.7%); Radical cystectomy: 5/58 (8.6%); Partial cystectomy: 1/58 (1.7%) |
|  | Center 2 | T1, Progression | TURBT: 28/38 (73.7%); Radical cystectomy: 10/38 (26.3%) |
|  | Center 1 | TURBT-to-TURBT balance | Progression 25/35 vs non-progression 44/51; Fisher's exact P = 0.105 |
|  | Center 2 | TURBT-to-TURBT balance | Progression 27/38 vs non-progression 48/58; Fisher's exact P = 0.211 |
| **Pre/post-intercurrent-treatment status** | Center 1 | Chronological status | For 81/86 treated pairs, T0 preceded and T1 followed the documented intercurrent postoperative treatment; 5/86 pairs had no documented intercurrent treatment and were coded separately. |
|  | Center 2 | Chronological status | For 90/96 treated pairs, T0 preceded and T1 followed the documented intercurrent postoperative treatment; 6/96 pairs had no documented intercurrent treatment and were coded separately. |
| **Interval/treatment/procedure-adjusted association** | Center 1 | Δ equivalent nuclear diameter mean; n = 86 | Adjusted OR per 1-SD feature = 1.12 (95% CI 0.67–1.85); P = 0.671. Interval OR per 1-SD = 0.62; P = 0.087. |
|  | Center 1 | Δ nuclear area mean; n = 86 | Adjusted OR per 1-SD feature = 1.36 (95% CI 0.81–2.26); P = 0.243. Interval OR per 1-SD = 0.58; P = 0.062. |
|  | Center 2 | Δ equivalent nuclear diameter mean; n = 96 | Adjusted OR per 1-SD feature = 2.58 (95% CI 1.50–4.45); P < 0.001. Interval OR per 1-SD = 1.42; P = 0.127. |
|  | Center 2 | Δ nuclear area mean; n = 96 | Adjusted OR per 1-SD feature = 2.50 (95% CI 1.45–4.32); P = 0.001. Interval OR per 1-SD = 1.39; P = 0.153. |
| **Restricted sensitivity analysis** | Center 1 | T0 intravesical chemotherapy only; 26/42 | Diameter: g = 0.05 (95% CI -0.44–0.54), P = 0.965; area: g = 0.20 (95% CI -0.29–0.69), P = 0.925 |
|  | Center 1 | TURBT-to-TURBT pairs; 25/44 | Diameter: g = -0.13 (95% CI -0.62–0.36), P = 0.481; area: g = 0.07 (95% CI -0.42–0.56), P = 0.591 |
|  | Center 1 | T0 intravesical + TURBT-to-TURBT; 22/38 | Diameter: g = -0.08 (95% CI -0.60–0.45), P = 0.629; area: g = 0.11 (95% CI -0.41–0.64), P = 0.718 |
|  | Center 1 | No documented intercurrent treatment; 4/1 | n = 5; too small for stable effect-size inference. |
|  | Center 2 | T0 intravesical chemotherapy only; 30/50 | Diameter: g = 0.70 (95% CI 0.23–1.17), P = 0.002; area: g = 0.66 (95% CI 0.19–1.12), P = 0.003 |
|  | Center 2 | TURBT-to-TURBT pairs; 27/48 | Diameter: g = 0.60 (95% CI 0.11–1.08), P = 0.010; area: g = 0.56 (95% CI 0.08–1.04), P = 0.012 |
|  | Center 2 | T0 intravesical + TURBT-to-TURBT; 23/42 | Diameter: g = 0.59 (95% CI 0.08–1.11), P = 0.021; area: g = 0.54 (95% CI 0.02–1.05), P = 0.025 |
|  | Center 2 | No documented intercurrent treatment; 3/3 | n = 6; too small for stable effect-size inference. |
| Abbreviations: BCG, Bacillus Calmette–Guérin; CI, confidence interval; IQR, interquartile range; MIBC, muscle-invasive bladder cancer; OR, odds ratio; TURBT, transurethral resection of bladder tumor; Δ, T1 minus T0. The T0–T1 interval was defined by the recorded T0 recurrence-free interval, which represented the time from the T0 operation/specimen to the recurrence/resection generating T1. T0 postoperative treatment was treated as the documented intercurrent exposure after T0 and before T1; T1 postoperative treatment occurred after acquisition of the T1 specimen and was not used as a pre-T1 exposure covariate. Surgical records were reported as surgery/procedure type. Adjusted logistic models included the standardized nuclear-size Δ feature, standardized T0–T1 interval, age, sex, baseline MIBC status, T0 intravesical-treatment exposure, and T0 TURBT status. Restricted analyses were interpreted as sensitivity analyses rather than independent validation. | | | |

**Figure S1. Extended longitudinal drift analysis in the Center 1 discovery cohort.** (A) Standardized mean differences of the top progression-associated pair-level Δ features in Center 1. Orange bars indicate positive effects in progression pairs, and blue bars indicate negative effects. Nominal *P* values and FDR-adjusted *P* values are shown for each feature. (B) Heatmap of selected Δ features across Center 1 paired cases ordered by progression status. Feature values were standardized for visualization. The dashed vertical line separates non-progression and progression pairs. (C) Paired T0-to-T1 trajectories of representative local-cellularity/spatial-organization and nuclear-size variability features stratified by progression status. (D) Group comparisons of selected Δ features between non-progression and progression pairs. Boxplots show pair-level Δ values with individual data points overlaid. (E) Outcome-informed exploratory longitudinal drift score in Center 1. Because feature screening and direction alignment used the progression label, this panel is retained only as a descriptive visualization and is not interpreted as independent confirmatory evidence. Δ indicates T1 minus T0; SMD, standardized mean difference; FDR, false discovery rate.

**Figure S2. Additional longitudinal drift patterns in the Center 2 confirmation cohort.** (A) Composition of progression and non-progression pairs in Center 2. (B) Heatmap of selected progression-associated pair-level Δ features across paired cases, ordered by progression status. Values were standardized for visualization; the dashed line separates non-progression and progression pairs. (C) Paired T0-to-T1 trajectories of representative nuclear size- and shape-related features in non-progression and progression groups. Thin lines denote individual pairs, and thick black lines denote group mean trajectories. Δ indicates T1 minus T0.

**Figure S3. Sensitivity analyses of progression-associated longitudinal drift.** (A) QC-enriched sensitivity analysis of key pair-level Δ features in Center 1 and Center 2. (B) Progression-definition sensitivity analysis under broad, strict stage-or-grade, and strict stage-only progression definitions. Points indicate standardized mean differences comparing progression with non-progression pairs; the dashed line denotes zero effect. Δ indicates T1 minus T0. (C) Tumor-region sensitivity analysis comparing whole-tissue and pathologist-confirmed tumor-only estimates for mean nuclear area and mean equivalent nuclear diameter in Centers 1 and 2. Points indicate standardized mean differences (Hedges' g), and horizontal bars indicate 95% confidence intervals; the dashed vertical line denotes zero effect. (D) Center 2 sensitivity analysis of mean nuclear area and mean equivalent nuclear diameter under broad (combined) progression, stage-only progression, NMIBC-to-MIBC transition, and grade-only upgrading definitions. Points indicate Hedges' g, horizontal bars indicate 95% confidence intervals, and the dashed vertical line denotes zero effect. Δ indicates T1 minus T0.

**Figure S4. Cross-backbone comparison of static WSI models.** (A,B) Center 1 internal pathology-only and fusion ROC curves. (C,D) Center 2, (E,F) Center 3, and (G,H) Center 4 locked external ROC curves. Models included clinical-only, UNI2, CTransPath, ResNet50, and DenseNet121 representations. AUCs with 95% confidence intervals are shown in the legends.

**Figure S5. Subgroup and time-dependent survival analyses of WSI-based risk scores.** (A) Time-dependent ROC curves for the clinical-only model and fusion models based on UNI2, CTransPath, ResNet50, and DenseNet121 in the Center 1 internal cohort at 36 and 60 months. (B) Kaplan–Meier analyses of UNI2 fusion risk groups in selected clinically relevant subgroups from Center 1, including NMIBC, high-grade disease, recurrent disease, and age ≥65 years. (C) Time-dependent ROC curves for the corresponding Center 1-derived locked models applied to Center 2 at 36 and 60 months. (D) Kaplan–Meier analyses of locked external UNI2 fusion risk groups in Center 2 using the fixed Center 1-derived cutoff, shown for the same clinically relevant subgroups. RFS, recurrence-free survival; ROC, receiver operating characteristic; WSI, whole-slide image.

**Figure S6. Extended TCGA molecular anchoring of pathology-derived axes.** (A–D) Heatmaps of top axis-associated genes in TCGA-BLCA, with patients ordered by the corresponding pathology-derived axis score: nuclear size axis, shape irregularity axis, spatial heterogeneity axis, and composite morphology axis, respectively. (E) Hallmark over-representation analysis of genes positively associated with the spatial heterogeneity axis. (F) Hallmark over-representation analysis of genes negatively associated with the composite morphology axis. Dot position indicates log2 odds ratio, dot size reflects enrichment strength, and color denotes log2 odds ratio. ORA, over-representation analysis; TCGA-BLCA, The Cancer Genome Atlas bladder urothelial carcinoma cohort.

**Figure S7. Extended exploratory IHC anchoring analyses.** (A) Association between ΔKi-67 index and the nuclear size-related drift axis. (B–C) Associations of T1 Ki-67 index with pair-level changes in mean equivalent nuclear diameter and mean nuclear area. (D) Distribution of the nuclear size-related drift axis across ordinal HER2 immunohistochemistry scores. These analyses were exploratory and were used to assess tissue-level concordance between nuclear size-related drift and immunohistochemical markers.
